# METTL3-mediated m6A modification of OTUD1 aggravates press overload induced myocardial hypertrophy by deubiquitinating PGAM5

**DOI:** 10.7150/ijbs.95707

**Published:** 2024-09-09

**Authors:** Kai Huang, Xiaotian Sun, Xiangyang Xu, Jie Lu, Boyao Zhang, Qin Li, Chuyi Wang, Sufan Ding, Xiaolei Huang, Xiaohong Liu, Zhiyun Xu, Lin Han

**Affiliations:** 1 Department of Cardiovascular Surgery, Changhai Hospital, Second Military Medical University, Shanghai, China.; 2 Department of Cardiothoracic Surgery, Huashan Hospital of Fudan University, Shanghai, China.; 3 Cardiac and Vascular Biology laboratory, Clinical and Translational Medicine Center, Changhai Hospital, Second Military Medical University, Shanghai, China.; 4 Institute of Thoracic Cardiac Surgery, Chinese People's Liberation Army, China.; 5 Key Laboratory of Cardiac Surgery, Chinese People's Liberation Army, China.; 6 Department of Pathology, Changhai Hospital, Second Military Medical University, Shanghai, China.

## Abstract

**Background**: Pathological cardiac hypertrophy, a condition that contributes to heart failure, is characterized by its intricate pathogenesis. The meticulous regulation of protein function, localization, and degradation is a crucial role played by deubiquitinating enzymes in cardiac pathophysiology. This study clarifies the participation and molecular mechanism of OTUD1 (OTU Deubiquitinase 1) in pathological cardiac hypertrophy.

**Methods**: We generated a cardiac-specific Otud1 knockout mouse line (Otud1-CKO) and adeno-associated virus serotype 9-Otud1 mice to determine the role of Otud1 in cardiac hypertrophy. Its impact on cardiomyocytes enlargement was investigated using the adenovirus. RNA immunoprecipitation was used to validate the specific m6a methyltransferase interacted with OTUD1 transcript. RNA sequencing in conjunction with immunoprecipitation-mass spectrometry analysis was employed to identify the direct targets of OTUD1. A series of depletion mutant plasmids were constructed to detect the interaction domain of OTUD1 and its targets.

**Results**: Ang II-stimulated neonatal rat cardiac myocytes and mice hearts subjected to transverse aortic constriction (TAC) showed increased protein levels of Otud1. Cardiac hypertrophy and dysfunction were less frequent in Otud1-CKO mice during TAC treatment, while Otud1 overexpression worsened cardiac hypertrophy and remodeling. METTL3 mediated m6A modification of OTUD1 transcript promoted mRNA stability and elevated protein expression. In terms of pathogenesis, Otud1 plays a crucial role in cardiac hypertrophy by targeting Pgam5, leading to the robust activation of the Ask1-p38/JNK signal pathway to accelerate cardiac hypertrophy. Significantly, the pro-hypertrophy effects of Otud1 overexpression were largely eliminated when Ask1 knockdown.

**Conclusion**: Our findings confirm that targeting the OTUD1-PGAM5 axis holds significant potential as a therapeutic approach for heart failure associated with pathological hypertrophy.

## Introduction

Cardiac hypertrophy is a physiological adaptation of the myocardium to the heightened mechanical workload induced by diverse pathological conditions 1, 2. Compensatory myocardial hypertrophy initially aids in augmenting cardiac output during the early phase of heart failure. Nevertheless, long-lasting pathological cardiac enlargement gradually results in decompensation, ultimately leading to irreversible cardiac dysfunction. The pathogenesis of myocardial hypertrophy (which involves substantial alterations in protein production, cellular size, contractile ability, collagen production, and fibrosis) is intricate 3. Previous studies have confirmed that mechanical stimulation, oxidative stress, and inflammation response are the main factors responsible for cell dysfunction in cardiac hypertrophy 4-6. Despite the extensive body of literature on this topic, there is currently a paucity of approved pharmacological interventions for cardiac hypertrophy. Consequently, identifying specific regulatory targets for myocardial hypertrophy holds great potential for developing novel therapeutic approaches to combat this disease.

Ubiquitination, an essential form of posttranslational modification, has a substantial impact on diverse cellular processes 7-10. Given its reversible nature, ubiquitination necessitates the involvement of deubiquitinating enzymes (DUBs) to counter-regulate its effects. Consequently, DUBs are indispensable for the precise regulation of protein function, localization, and deregulation, thereby exerting a crucial influence on cellular signaling networks and pathophysiology 11, 12.

The OTU (ovarian tumor-related proteases) family members exhibit an Alanine-rich region (Ala), a catalytic OTU domain (OTU), and a ubiquitin-interacting motif (UIM) 13. The OTU family is associated with several pathological processes, including protein degradation, autophagy regulation, immune response, and tumorigenesis 14-19. However, the role of OTU family members in cardiac hypertrophy-related heart failure requires further exploration.

One study reported that OTUD1 regulated pathological vascular remodeling 20. OTUD1 deletion attenuates vascular remodeling, collagen deposition, and endothelial-to-mesenchymal transition. Bioinformatics analyses also confirmed OTUD1 upregulation expression in cardiomyocytes after pathological stimulation 21, 22. Nevertheless, there is still a gap in understanding the comprehensive regulatory network of OTUD1 in the context of controlling cardiac hypertrophy and remodeling.

N6-methyladenosine (m6A) is the most studied RNA modification and controls multiple cellular functions in cardiovascular disease. Previous studies revealed the increased m6A modification in myocardium under hypertrophic stimulation and showed the possibility of the involvement of m6A in the regulation of cardiomyocyte homeostasis *in vivo* 23-25.

This study revealed an increase in the protein expression of Otud1 in cardiomyocytes stimulated by Ang II and in the myocardium induced by transverse aortic constriction (TAC) owing to the m6A modification on transcript of OTUD1. Otud1 overexpression exacerbated cardiac hypertrophy and dysfunction, while its deficiency alleviated pathological cardiac hypertrophy. Mechanistically, OTUD1 facilitated the procession of cardiac hypertrophy by directly binding to and deubiquitinating PGAM5 in a manner dependent on K63 ubiquitin chains, leading to enhanced ASK1 phosphorylation and p38/JNK MAPK signal pathway activation in cardiomyocytes. We identified OUTD1 as a new regulator of the MAPK signal pathway and myocardial hypertrophy.

## Methods

Data availability, RNA sequencing access number, experimental methods, mice models, and cell culture are described in the Supplemental Material.

For cardiac-specific Otud1 knockout mice, the Otud1-Flox mice (Cyagen Biosciences, Guangzhou, China) were crossed with ɑ-MHC-MerCreMer transgenic mice to obtained Otud1^loxP/loxP^/ɑ-MHC-MCM mice. Then we quickly obtained enough mice through *in vitro* fertilization and embryo transfer. The cardiomyocyte specific conditional Otud1 gene knockout (Otud1-CKO) mice were induced through 6-week-old Otud1^loxP/loxP^/ɑ-MHC-MCM mice being injected with tamoxifen for 5 days. Otud1-floxed mice were also treated with equal doses of tamoxifen injection as the controls.

Antibodies, primer sequences for PCR analyses and plasmid construction are shown in **Tables S1-S4**. The data, analytical methods, and study materials are available from the corresponding author upon reasonable request.

## Results

### The upregulation of OTUD1 in cardiac hypertrophy

**Figure 1:**
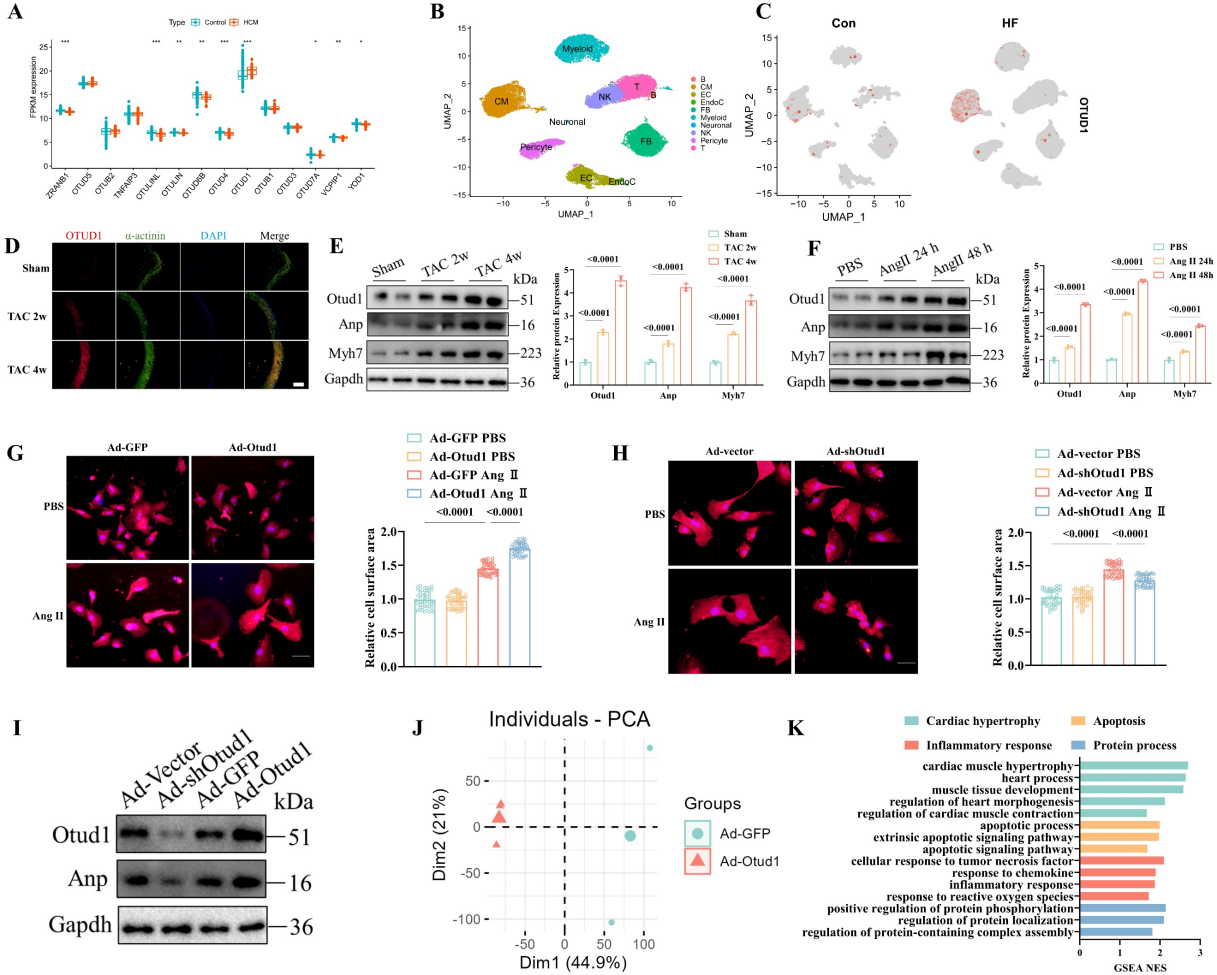
OTUD1 is up-regulated during pathological cardiac hypertrophy. (A) Boxplot showing the expression of genes from OUT family in GSE141910. (B) Single cell analysis showing the UMAP plot of cell distribution in heart failure. (C) Single cell analysis showing the upregulation of OTUD1 in cardiomyocytes from heart failure samples. (D) Otud1 protein levels in heart tissues were determined by immune fluorescence. Scale bar, 100 μm. (E) Otud1 protein levels in heart tissues were determined by western blot. Quantification of the relative OTUD1 protein level (right; n= 3). (F) Otud1 protein levels in NRCMs were determined by western blot. Quantification of the relative OTUD1 protein level (right; n= 3). (G) Representative immunofluorescence images of α-actinin staining of NRCMs infected with Ad-GFP or Ad-Otud1 and treated with AngⅡ (10 μM) or PBS for 48h. Scale bar, 50 μm. Quantitative results of the cell surface area of NRCMs (right, n ≥ 50 cells per group). (H) Representative immunofluorescence images of α-actinin staining of NRCMs infected with Ad-vector or Ad-shOtud1 and treated with AngⅡ (10 μM) or PBS for 48h. Scale bar, 50 μm. Quantitative results of the cell surface area of NRCMs (right, n ≥ 50 cells per group). (I) Representative western blotting results of Anp in NRCMs transfected with Ad-Otud1 or Ad-shOtud1 (n=3). (J) PCA showing the global sample distribution profiles between Ad-GFP and Ad-Otud1 based on the RNA-seq data. (K) GESA of biological processes involved in cardiac hypertrophy, Apoptosis, inflammatory response and protein process in RNA-seq data.

We initially examined the OTU family's deubiquitinating enzymes in public cardiac hypertrophy and heart failure datasets. In the cardiac hypertrophy dataset, GSE141910, we discovered that OTUD1 was the sole upregulated OTU member (**Figure 1A**). In the GSE57338 dataset (one heart failure dataset), both OTUD1 and TNFAIP3 were upregulated (**Figure S1A**). Moreover, the higher expression of OTUD1 protein was validates in human cardiac tissues (**Figure S1B**). Consequently, OTUD1 was selected for further analyses. Afterward, we examined the cellular distribution of OTUD1 at the single-cell level. Three human single-cell sequencing datasets (GSE161470, GSE145154, and GSE161153) were integrated, comprising four control samples and four heart failure samples 26-28. Our analyses confirmed that OTUD1 was predominantly detected in cardiomyocytes, with heart failure samples demonstrating significantly higher levels of OTUD1 expression compared to control samples (**Figure 1B-C**). The cell distribution of OTUD1 was also validated by western blot, showing the cardiomyocytes was the main enriched cell, other than fibroblasts (**Figure S1C**). Hence, we speculated that OTUD1 could regulate the development of cardiac hypertrophy and heart failure. OTUD1 upregulation was confirmed by immunofluorescence staining (**Figure 1D**) and western blot (**Figure 1E**) in TAC mice. We also found that OTUD1 protein expression was elevated in Ang II-stimulated neonatal rat cardiac myocytes (NRCMs) (**Figure 1F**).

Then, Otud1-expressing adenovirus (Ad-Otud1) and shRNA targeting Otud1 (Ad-shOtud1) were constructed to infect NRCMs. **Figure 1G** demonstrate that Ad-Otud1 increased the cell surface of NRCMs, while the cellular surface of cardiomyocytes was significantly reduced in the Ad-shOtud1 cell group (**Figure 1H**). Similarly, the protein expression of Anp increased after Otud1 overexpression, and this was reversed by Otud1 knockdown (**Figure 1I**).

To elucidate the regulatory mechanism of Otud1 in cardiac hypertrophy, RNA sequencing analyses of NRCMs infected with Ad-GFP and Ad-Otud1 were performed. The principal component analysis demonstrated that the transcriptome profile was separated into two clusters (**Figure 1J**). Gene set enrichment analyses revealed that genes regulated by Otud1 were enriched in myocardial hypertrophy, apoptosis, inflammatory response, and protein processes (**Figure 1K**). The heatmaps also demonstrated that OTUD1 overexpression significantly elevated the expression of genes involved in the biological processes mentioned above (**Figure S1B-E**). These results demonstrated the pro-hypertrophy effect of Otud1 on Ang II-stimulated NRCMs.

### OTUD1 knockout alleviates TAC-induced Cardiac Hypertrophy in Mice

**Figure 2:**
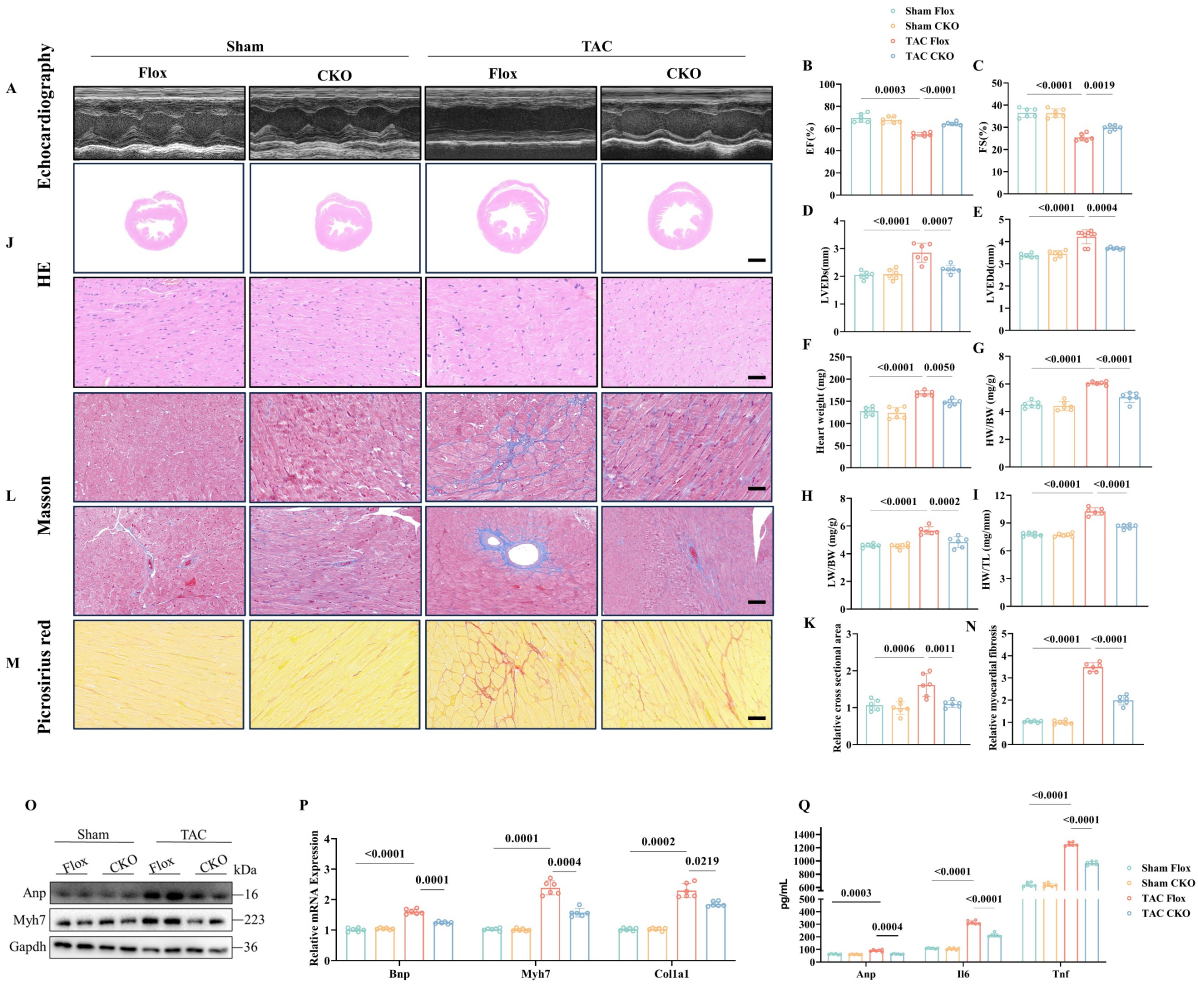
Otud1 knockout alleviates transverse aortic constriction (TAC)-induced cardiac hypertrophy *in vivo*. (A) Representative M-mode echocardiographic images from each group in mice (n=6). (B) Assessments of ejection fraction (EF) in Flox and CKO mice at 4 weeks after Sham or TAC surgery (n=6). (C) Assessments of fraction shortening (FS) in Flox and CKO mice at 4 weeks after Sham or TAC surgery (n=6). (D) Assessments of left ventricular end-systolic dimension (LVEDs) in Flox and CKO mice at 4 weeks after Sham or TAC surgery (n=6). (E) Assessments of left ventricular (LV) end-diastolic dimension (LVEDd) in Flox and CKO mice at 4 weeks after Sham or TAC surgery (n=6). (F) Assessments of heart weight (HW) in Flox and CKO mice at 4 weeks after Sham or TAC surgery (n=6). (G) Assessments of HW/body weight (BW) ratios in Flox and CKO mice at 4 weeks after Sham or TAC surgery (n=6). (H) Assessments of lung weight (LW)/BW ratios in Flox and CKO mice at 4 weeks after Sham or TAC surgery (n=6). (I) Assessments of HW/tibia length (TL) ratios in Flox and CKO mice at 4 weeks after Sham or TAC surgery (n=6). (J) Representative images of hematoxylin-eosin (HE) staining of LV cross sections in the hearts of Flox and CKO mice at 4 weeks after Sham or TAC surgery (n=6). Scale bar, 1000 μm for the top set and 50 μm for the bottom parts. (K) Relative cross-sectional areas from the hearts of in Flox and CKO mice at 4 weeks after Sham or TAC surgery (n=6). (L) Representative images of Masson staining of cross-sections in the hearts of in Flox and CKO mice at 4 weeks after Sham or TAC surgery (n=6). Scale bar, 50 μm. (M) Representative images of picrosirius red staining of cross-sections in the hearts of Flox and CKO mice at 4 weeks after Sham or TAC surgery (n=6). Scale bar, 50 μm. (N) Relative cardiac fibrosis from the hearts of Flox and CKO mice at 4 weeks after Sham or TAC surgery (n=6). (O) Representative western blotting results of Myh7 and Anp in hearts from Flox and CKO mice at 4 weeks after Sham or TAC surgery. The experiment was repeated three times. (P) Real-time qPCR analysis of Bnp, Myh7, Col1a1 in heart tissues from Flox and CKO mice at 4 weeks after Sham or TAC surgery (n=6). (Q) The content of Anp, Il-6, Tnf-a in plasma from Flox and CKO mice at 4 weeks after Sham or TAC surgery (n=6).

To further investigate the involvement of Otud1 in the development of pathological cardiac hypertrophy *in vivo*, we utilized Otud1-CKO mice (**Figure S2A**). After four weeks following TAC surgery, Flox and CKO mice were subjected to additional analyses. Echocardiography revealed that Otud1-CKO mice exhibited significant preservation in terms of the ejection fraction, fraction shortening (FS), left ventricular end-systolic diameter, and left ventricular end-diastolic diameter (LVEDd) compared to their Flox counterparts (**Figure 2A-E**). Compared to Flox mice, Otud1-CKO mice exhibited reduced heart weight (HW), heart weight/body weight (HW/BW), lung weight/body weight (LW/BW), and heart weight/tibia length ratios (HW/TL) (**Figure 2F-I**). Additionally, in the TAC model, Otud1-CKO mice displayed a smaller cross-sectional area of cardiomyocytes (**Figure 2J-K**). Moreover, Otud1 deficiency exhibited the potential to mitigate TAC-induced cardiac fibrosis, as demonstrated by the utilization of Masson and Picrosirius Red staining (**Figure 2L-N**). The protein expression of genes associated with cardiac hypertrophy (Anp and Myh7), was also found to be reduced in mice lacking Otud1 (**Figure 2O**). The levels of Bnp, Myh7, and Col1a1 mRNA expression were observed to be elevated in WT mice following TAC; however, this trend was reversed in CKO mice (**Figure 2P**). Moreover, Otud1-CKO also prevented the elevation of serum Anp, Il-6, and Tnf-a (**Figure 2Q**). In summary, the absence of Otud1 successfully mitigated the development of pathological cardiac hypertrophy and cardiac fibrosis caused by TAC.

We also introduced Otud1-specific overexpression in cardiomyocytes using AAV9 to strengthen the results presented above (**Figure S2B**). The effectiveness of Otud1 overexpression was assessed through western blot analyses (**Figure S2C**). In contrast to the findings in Otud1-CKO mice, AAV9-Otud1 administration effectively promoted TAC-related cardiac dysfunction (**Figure S3**).

### m6A modification leads to OTUD1 upregulation in cardiac hypertrophy

**Figure 3:**
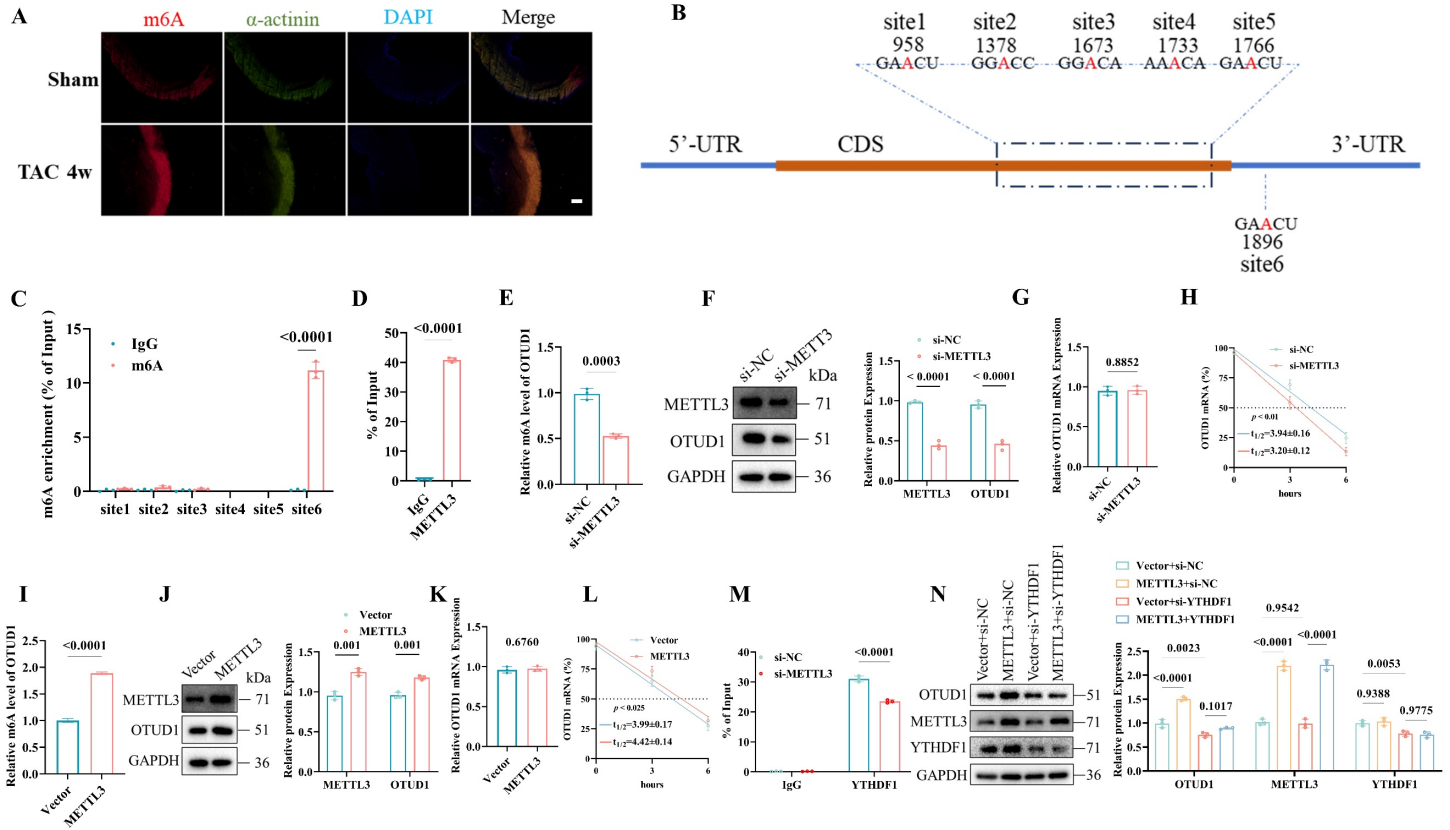
OTUD1 is up-regulated during pathological cardiac hypertrophy due to m6A modification. (A) m6A levels in heart tissues were determined by immune fluorescence. Scale bar, 100 μm. (B) Schematic diagram showing the position of m6A motifs within OTUD1 transcript sequence. (C) m6A RIP analysis of m6A levels of OTUD1 at different sites in AC16 (n=3). (D) The interaction between the METTL3 protein and OTUD1 mRNA in AC16 was measured by RNA immunoprecipitation followed by qRT-PCR analysis (n=3). (E) The m6A levels of OTUD1 transcript after si-METTL3 transfection in AC16 (n=3). (F) OTUD1 protein levels in AC16 transfected with si-METTL3. Quantification of the relative protein level (right). The experiment was repeated three times. (G) The mRNA levels of OTUD1 after si-METTL3 transfection in AC16 (n=3). (H) OTUD1 RNA stability in METTL3-knockdown AC16 was measured by one-phase decay analysis (n=3). (I) The m6A levels of OTUD1 transcript after METTL3 plasmid transfection in AC16 (n=3). (J) OTUD1 protein levels in AC16 transfected with METTL3 plasmid. Quantification of the relative protein level (right). The experiment was repeated three times. (K) The mRNA levels of OTUD1 after METTL3 plasmid transfection in AC16 (n=3). (L) OTUD1 RNA stability in METTL3 overexpression AC16 was measured by one-phase decay analysis (n=3). (M) The interaction between the YTHDF1 protein and OTUD1 mRNA in METTL3-knockdown AC16 was measured by RIP followed by qRT-PCR analysis (n=3). (N) Western blot analysis of OTUD1 protein levels in AC16 after transfection with or without METTL3 plasmid, followed by treatment with or without si-YTHDF1. Quantification of the relative protein level (right). The experiment was repeated three times.

Previous studies have indicated that the N6-methyladenosine (m6A) modification might be one new regulator of pathological cardiac hypertrophy 25, 29. Immunofluorescence staining confirmed that high m6A (**Figure 3A**) and METTL3 (**Figure S4A**) levels were induced in murine hearts by transverse aortic constriction (TAC). The m6A dot blot showed that Ang II treatment could elevate the global m6A in cardiomyocytes and METTL3 overexpression could upregulate the m6A level (**Figure S4B**). To clarify the relationship between METTL3-mediated m6A modification and OTUD1 upregulation, we used one online tool 30 and identified five m6A modifications within the coding sequence region and one m6A site in the 3′-untranslated region (**Figure 3B, Figure S4C**). Our m6A RIP analysis revealed significant enrichment of m6A modifications at site 6 within the OTUD1 transcript (**Figure 3C**). The relative enrichment of m6A-modified OTUD1 mRNA was substantially higher in the samples immunoprecipitated with the anti-m6A antibody compared to the IgG control, confirming the presence of m6A modifications at this specific site. These findings support the notion that METTL3-mediated m6A modification enhances the stability and expression of OTUD1 mRNA. METTL3 could directly interact with OTUD1 mRNA (**Figure 3D**). Then, we transfected the human cardiomyocyte cell line (AC16) with si-METTL3 or METTL3 plasmids. METTL3 knockdown reduced m6A level of OTUD1 transcript (**Figure 3E**) and OTUD1 protein levels (**Figure 3F**) and its mRNA half-life (**Figure 3H**). While, the mRNA expression of OTUD1 was not altered under si-METTL3 (**Figure 3G**). In contrast, METTL3 overexpression resulted in elevated m6A level of OTUD1 transcript (**Figure 3I**), OTUD1 protein levels (**Figure 3J**), mRNA half-life of OTUD1 (**Figure 3L**) and unchanged mRNA expression of OTUD1 (**Figure 3K**). These results suggested that m6A modification facilitated by METTL3 could promote OTUD1 protein expression by enhancing its mRNA stability.

The YTH domain family (YTHDF1, YTHDF2, YTHDF3) serves as “readers” to recognize the m6A-modified mRNA. To figure out which binding protein selectively regulated METTL3-modified OTUD1 mRNA, we transfected AC16 with si-YTHDF1, si-YTHDF2, and si-YTHDF3. It was demonstrated that YTHDF1 knockdown (**Figure S4D**), and not YTHDF2 (**Figure S4E**) or YTHDF3 (**Figure S4F**) knockdown, alleviated OTUD1 protein expression. The mRNA half-time was also reduced due to YTHDF1 knockdown (**Figure S4G**). In contrast, YTHDF1 overexpression increased OTUD1 protein levels (**Figure S4H**) and the mRNA half-life of OTUD1 (**Figure S4I**). Moreover, YTHDF1 had a direct interaction with OTUD1 mRNA (**Figure S4J**), which was reduced or strengthened by the knockdown (**Figure 3M**) or overexpression (**Figure S4K**) of METTL3, respectively. Hence, we reported that the m6A-modified mRNA of OTUD1 by METTL3 was a target of YTHDF1.

Next, we transfected the METTL3 plasmid and si-YTHDF1 in AC16 at the same time and demonstrated that the elevated protein expression of OTUD1 by METTL3 overexpression could be rescued by YTHDF1 silencing (**Figure 3N**). YTHDF1 overexpression could restore the decreased protein expression of OTUD1 caused by silencing METTL3 (**Figure S4L**).

**Figure 4:**
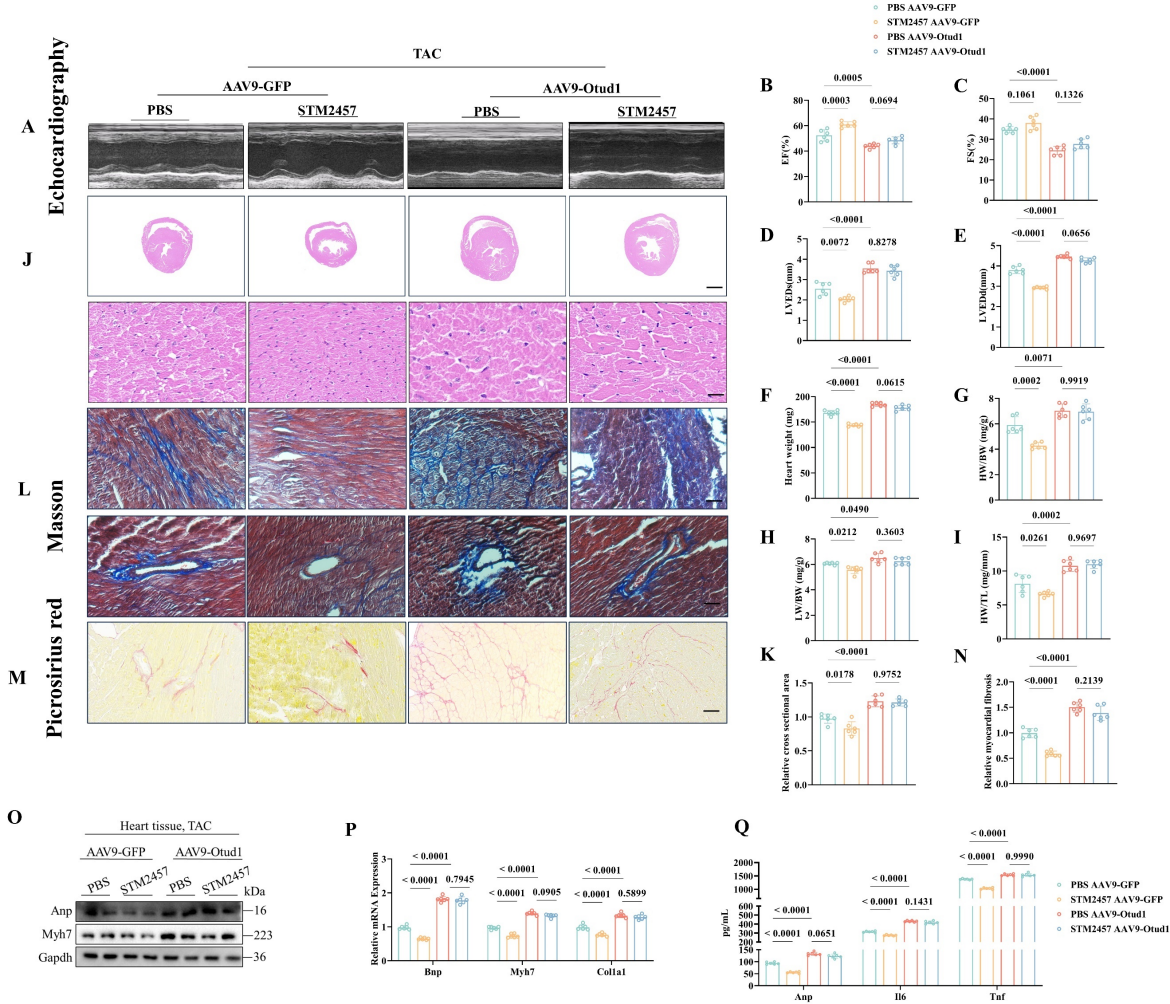
METTL3 promotes cardiac hypertrophy in an OTUD1 depend way. (A) Representative M-mode echocardiographic images from each group in mice (n=6). (B) Assessments of ejection fraction (EF) in PBS and STM2457 treated TAC mice injected with AAV9-GFP or AAV9-Otud1 at 4 weeks (n=6). (C) Assessments of fraction shortening (FS) in PBS and STM2457 treated TAC mice injected with AAV9-GFP or AAV9-Otud1 at 4 weeks (n=6). (D) Assessments of left ventricular end-systolic dimension (LVEDs) in PBS and STM2457 treated TAC mice injected with AAV9-GFP or AAV9-Otud1 at 4 weeks (n=6). (E) Assessments of left ventricular (LV) end-diastolic dimension (LVEDd) in PBS and STM2457 treated TAC mice injected with AAV9-GFP or AAV9-Otud1 at 4 weeks (n=6). (F) Assessments of heart weight (HW) in PBS and STM2457 treated TAC mice injected with AAV9-GFP or AAV9-Otud1 at 4 weeks (n=6). (G) Assessments of HW/body weight (BW) ratios in PBS and STM2457 treated TAC mice injected with AAV9-GFP or AAV9-Otud1 at 4 weeks (n=6). (H) Assessments of lung weight (LW)/BW ratios in PBS and STM2457 treated TAC mice injected with AAV9-GFP or AAV9-Otud1 at 4 weeks (n=6). (I) Assessments of HW/tibia length (TL) ratios in PBS and STM2457 treated TAC mice injected with AAV9-GFP or AAV9-Otud1 at 4 weeks (n=6). (J) Representative images of hematoxylin-eosin (HE) staining of LV cross sections in the hearts of PBS and STM2457 treated TAC mice injected with AAV9-GFP or AAV9-Otud1 at 4 weeks (n=6). Scale bar, 1000 μm for the top set and 50 μm for the bottom parts. (K) Relative cross-sectional areas from the hearts of PBS and STM2457 treated TAC mice injected with AAV9-GFP or AAV9-Otud1 at 4 weeks (n=6). (L) Representative images of Masson staining of cross-sections in the hearts of PBS and STM2457 treated TAC mice injected with AAV9-GFP or AAV9-Otud1 at 4 weeks (n=6). Scale bar, 50 μm. (M) Representative images of picrosirius red staining of cross-sections in the hearts of PBS and STM2457 treated TAC mice injected with AAV9-GFP or AAV9-Otud1 at 4 weeks (n=6). Scale bar, 50 μm. (N) Relative cardiac fibrosis from the hearts of PBS and STM2457 treated TAC mice injected with AAV9-GFP or AAV9-Otud1 at 4 weeks (n=6). (O) Representative western blotting results of Myh7 and Anp in hearts from PBS and STM2457 treated TAC mice injected with AAV9-GFP or AAV9-Otud1 at 4 weeks. The experiment was repeated three times. (P) Real-time qPCR analysis of Bnp, Myh7, Col1a1 in heart tissues from PBS and STM2457 treated TAC mice injected with AAV9-GFP or AAV9-Otud1 at 4 weeks (n=6). (Q) The content of Anp, Il-6, Tnf-a in plasma from PBS and STM2457 treated TAC mice injected with AAV9-GFP or AAV9-Otud1 at 4 weeks (n=6).

To further validate role of METTL3 mediated OTUD1 RNA modification in cardiac hypertrophy *in vivo*, we used METTL3 inhibitor (STM2457) to treat AAV9-Otud1 mice. STM2457 could effectively reduce the protein expression of METTL3 (**Figure S4M**). As showed in **Figure 4**, the protective effect of METTL3 inhibition could be rescued by OTUD1 overexpression to some extent. These findings proved that METTL3 specifically targeted OTUD1 transcript and facilitated the translation of m6A-methylated OTUD1 mRNA in a YTHDF1-dependent manner.

### OTUD1 activates the ASK1-p38/JNK pathways during cardiac hypertrophy

**Figure 5:**
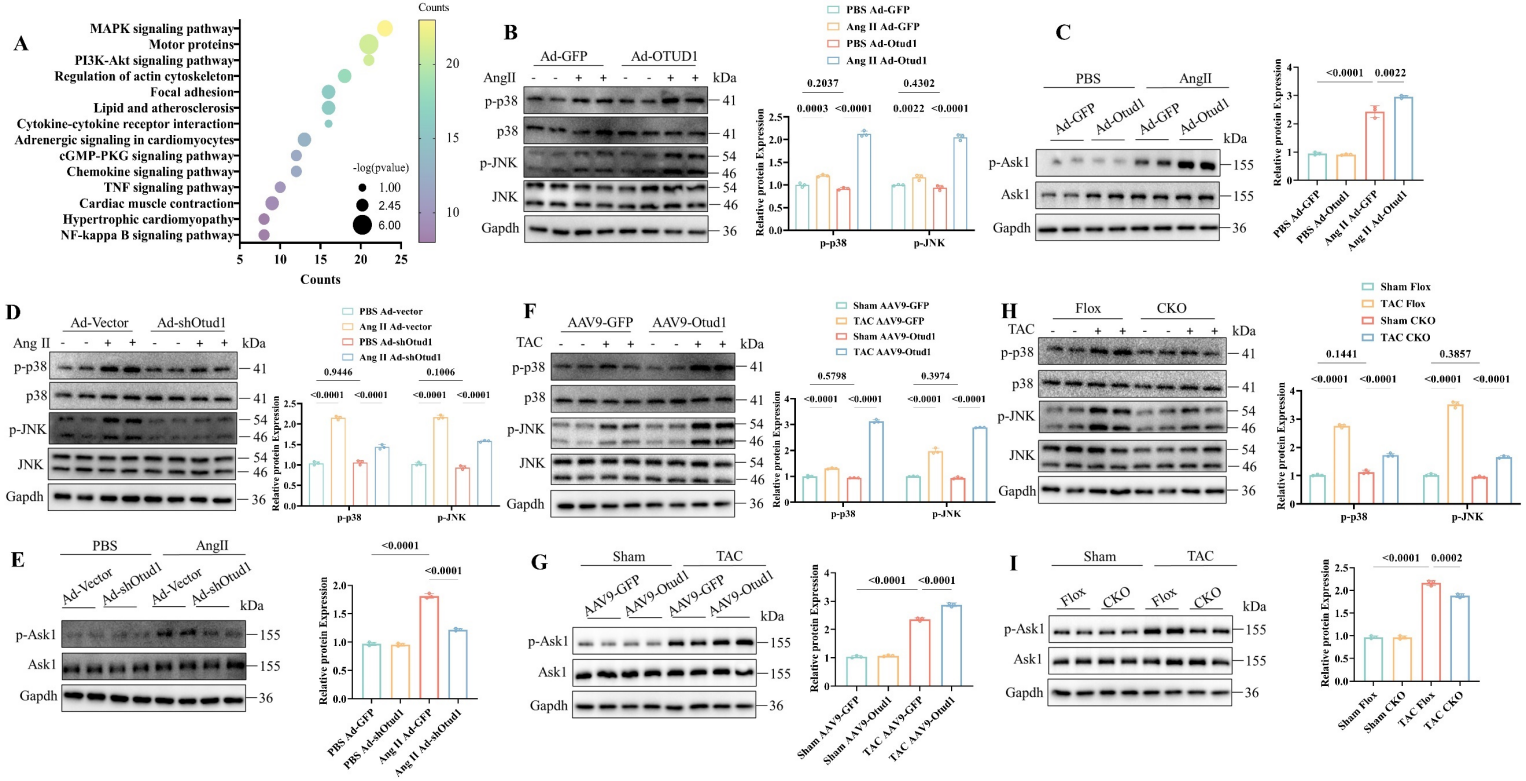
OTUD1 activates the ASK1-p38/JNK pathways during cardiac hypertrophy. (A) The significantly enriched pathways for the function of Otud1 were determined by KEGG enrichment analysis. (B) Immunoblotting analysis of p-p38, p38, p-JNK and JNK in NRCMs infected with Ad-Otud1 and stimulated with AngⅡ (10 μM) for 48h. Quantification of the relative protein level (right; n= 3). (C) Immunoblotting analysis of p-Ask1, Ask1 in NRCMs infected with Ad-Otud1 and stimulated with AngⅡ (10 μM) for 48h. Quantification of the relative protein level (right; n= 3). (D) Immunoblotting analysis of p-p38, p38, p-JNK and JNK in NRCMs infected with Ad-shOtud1 and stimulated with AngⅡ (10 μM) for 48h. Quantification of the relative protein level (right; n= 3). (E) Immunoblotting analysis of p-Ask1, Ask1 in NRCMs infected with Ad-shOtud1 and stimulated with AngⅡ (10 μM) for 48h. Quantification of the relative protein level (right; n= 3). (F) Immunoblotting analysis of p-p38, p38, p-JNK and JNK in hearts infected with AAV9-Otud1 and subjected to TAC for 4 weeks. Quantification of the relative protein level (right; n= 3). (G) Immunoblotting analysis of p-Ask1, Ask1 in hearts infected with AAV9-Otud1 and subjected to TAC for 4 weeks. Quantification of the relative protein level (right; n= 3). (H) Immunoblotting analysis of p-p38, p38, p-JNK and JNK in Otud1-CKO hearts subjected to TAC for 4 weeks. Quantification of the relative protein level (right; n= 3). (I) Immunoblotting analysis of p-Ask1, Ask1 in Otud1-CKO hearts subjected to TAC for 4 weeks. Quantification of the relative protein level (right; n= 3).

Subsequently, we aimed to investigate the underlying molecular mechanism by which Otud1 regulates cardiac hypertrophy. We conducted RNA sequencing between Otud1-overexpressed and control NRCMs and KEGG analysis revealed that differentially expressed genes were mostly enriched in the MAPK pathway (**Figure 5A**). We speculated that OTUD1 might promote cardiac hypertrophy by activating MAPK signaling. MAPK signaling is composed of four main molecules, including ERK1/2, ERK5, JNK and p38 31. The phosphorylation level of ERK1/2 and ERK5 showed no obvious changes in Otud1 overexpressed NVCMs (**Figure S5A**). We found that p38/JNK pathway was significantly activated in Otud1 overexpressed NRCMs exposed to Ang II treatment (**Figure 5B**). ASK1 is a member of the MAPK family, which can activate p38/JNK signaling. It was found that the phosphorylation levels of ASK1 were also significantly enhanced (**Figure 5C**), but the total levels of ASK1 and JNK/p38 did not change. Opposite results were observed in Ad-shOtud1 treated NRCMs (**Figure 5D-E**). *In vivo* experiments further proved the activation effect of OTUD1 on ASK1-p38/JNK pathway (**Figure 5F-I**). These results demonstrate that OTUD1 activates the ASK1-p38/JNK signaling pathway in response to pro-hypertrophy stimulation.

### OTUD1 binds to and deubiquitinates PGAM5

**Figure 6:**
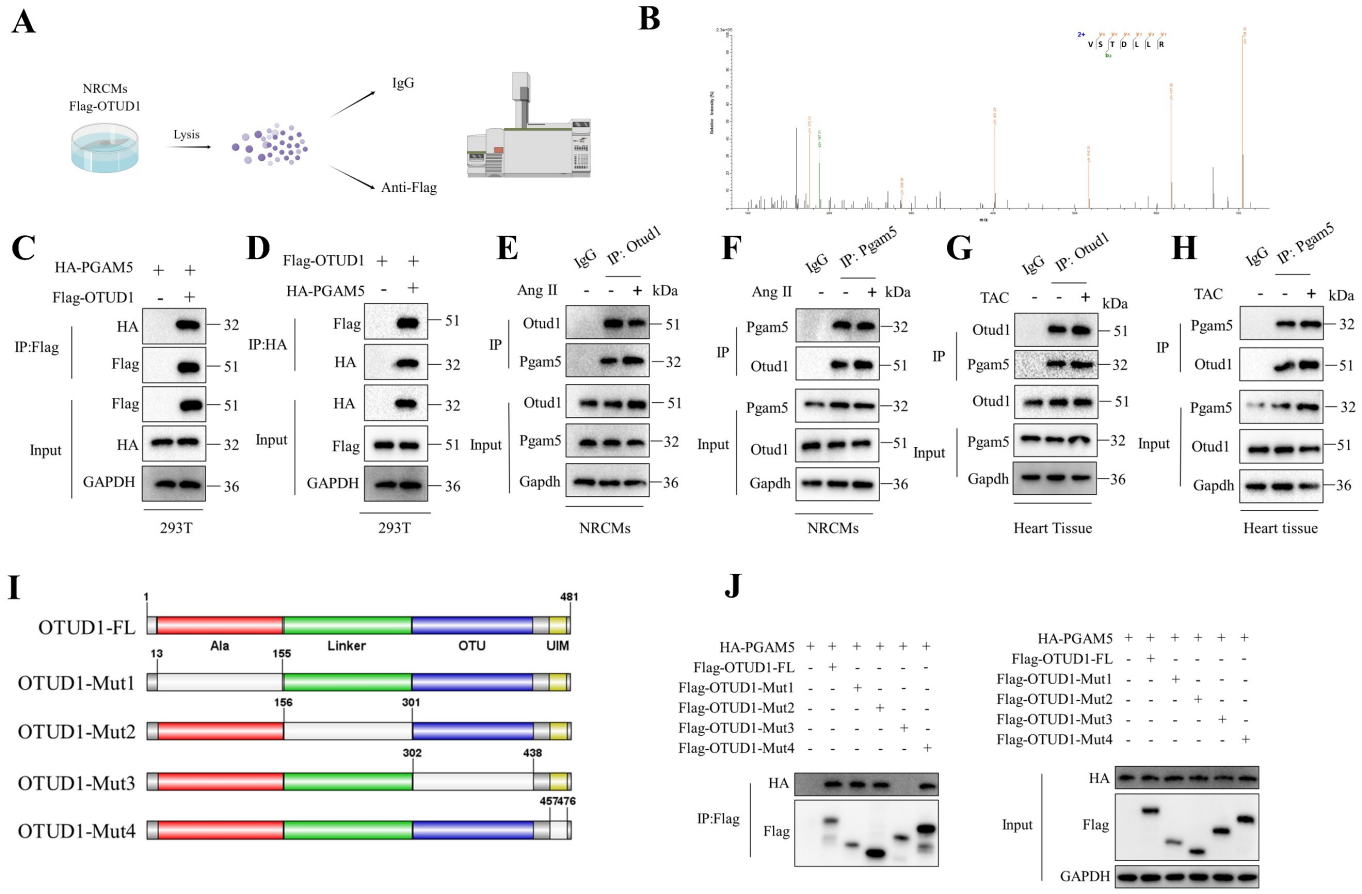
OTUD1 directly binds to PGAM5. (A) Schematic illustration of mass spectrometry analysis to identify proteins binding to OTUD1. (B) PGAM5 peptides derived from the mass spectrometric analysis of OTUD1. (C-D) Co-IP assays of the interaction between OTUD1 and PGAM5 in 293T cells transfected with the indicated plasmids. (E-F) Co-IP assays of Otud1 and Pgam5 in NRCMs. (G-H) Co-IP assays of Otud1 and Pgam5 in heart tissues. (I) Schematic illustration of the OTUD1 domain deletion mutants. (J) Co-IP analysis of the binding region of OTUD1 and PGAM5.

To further elucidate the mechanism by which OTUD1 regulates ASK1 activity, we conducted an Otud1 immunoprecipitation-mass spectrometry (MS) analysis (**Figure 6A**). Among the potential proteins binding to OTUD1, PGAM5 has piqued our interest due to its role as an upstream regulator for ASK1 phosphorylation (T845) and p38/JNK pathways and its reported involvement in ischemia reperfusion injury 32, 33. To test the role of PGAM5 in pathological cardiac hypertrophy, we used PGAM5 inhibitor LFHP-1c (HY-139598, MCE) in TAC mice. Inhibition of PGAM5 could alleviate cardiac hypertrophy (Figure S6A-L). **Figure 6B** displays the representative MS spectra of PGAM5. The initial confirmation of the interaction between OTUD1 and PGAM5 was conducted in 293T cells (**Figure 6C-D**). Subsequently, this interaction was found to be enhanced in NRCMs following stimulation with Ang II and TAC hearts (**Figure 6E-H**). OTUD1 consists of four domains: one Ala-rich domain (Ala), one linker domain (Linker), and one OTU domain (OTU). and one ubiquitin interacting motif (UIM) (**Figure 6I**). We then constructed four deletion mutants of OTUD1 and co-transfected these mutants and PGAM5 plasmids in 293T cells. The OTU domain of OTUD1 was necessary for its interaction with PGAM5 (**Figure 6J**). We employed adenoviral vectors to overexpress both the full-length OTUD1 and four OTUD1 mutants in NRVMs. Our findings indicated that the deletion of the OTU domain did not exhibit any pro-hypertrophic effects on the NRVMs (Figure S7), suggesting the critical role of OTU domain in OTUD1-induced cardiac hypertrophy.

**Figure 7:**
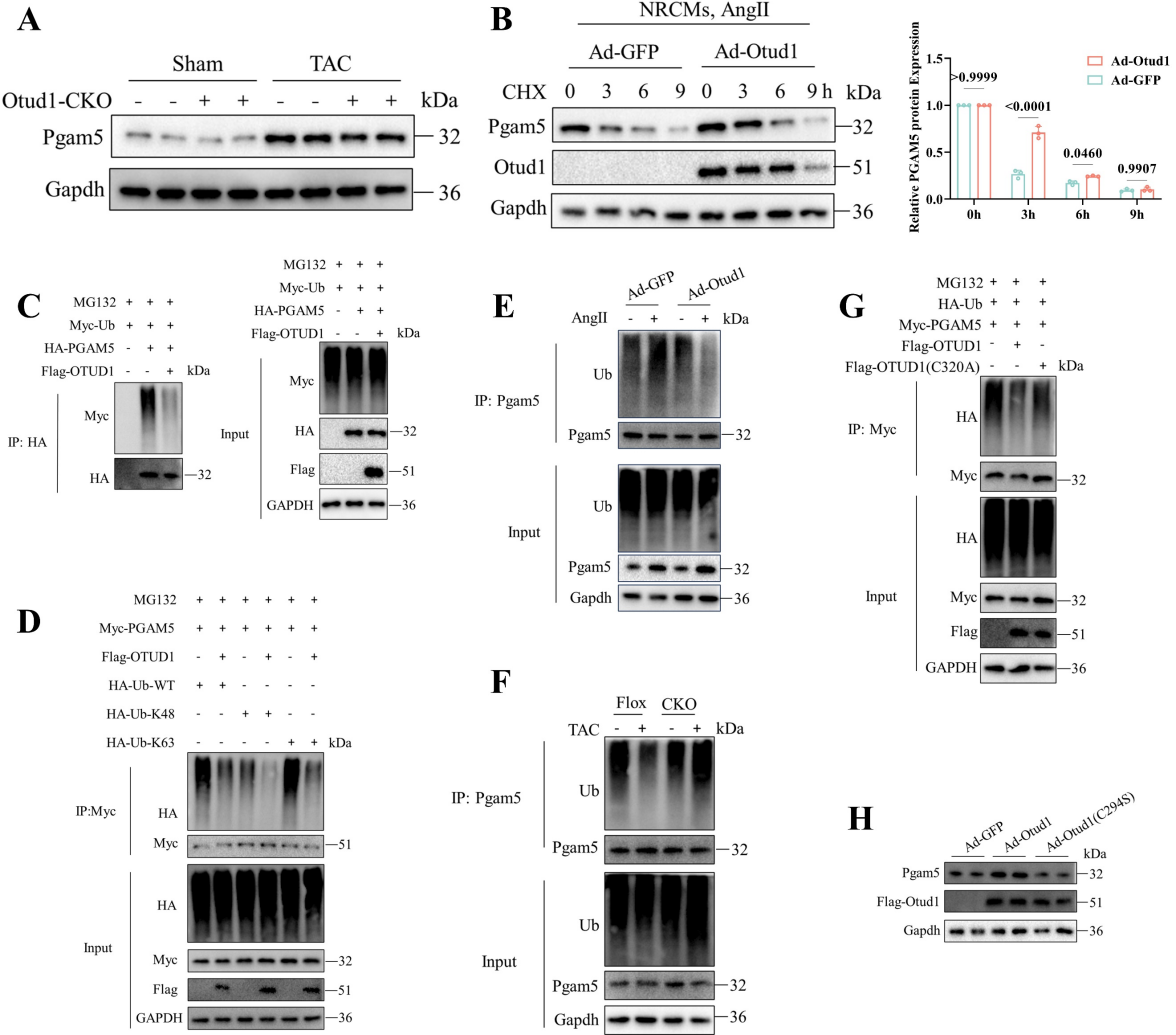
OTUD1 deubiquitinates PGAM5. (A) Representative western blot of Pgam5 protein in heart of Flox and Otud1-CKO mice subjected to Sham or TAC (n=3). (B) Representative western blotting result of Pgam5 in NRCMs infected with Ad-GFP and Ad-Otud1 and treated with Ang II (10 μM) for 48h and cycloheximide (CHX, 50 μM) for the indicated time points (n=3). (C) Results of ubiquitination assays confirming the ubiquitination of PGAM5 after overexpression of OTUD1 for 48 h and treated with MG132 (50 μM) for 6 h in 293T. (D) Results of ubiquitination assays confirming the K48 ubiquitination of PGAM5 after co-transfected with Flag-OTUD1, HA-Ub-WT, HA-Ub-K48, HA-Ub-K63 for 24 h and treatment with MG132 (50 μM) for 6 h in 293T. (E) Results of ubiquitination assays confirming the expression and ubiquitination of Pgam5 in NRCMs infected with Ad-GFP and Ad-Otud1 and followed by Ang Ⅱ treatment. (F) Results of ubiquitination assays confirming the expression and ubiquitination of Pgam5 in heart tissue from Flox and CKO mice subjected to sham or TAC. (G) Results of ubiquitination assays confirming the ubiquitination of Pham5 after overexpression of OTUD1, OTUD1(C320A) and Pgam5 for 24 h and treated with MG132 (50 μM) for 6 h in 293T cells. (H) Immunoblotting analysis of Pgam5, Ad-Otud1 and Ad-Otud1 (C294S) in NRCMs.

Given that DUBs play a role in the modulation of biological activities by influencing the degradation or function of substrate proteins, our subsequent investigation aimed to determine whether Otud1 could deubiquitinating pgam5. We observed decreased Pgam5 protein expression after TAC in Otud1-CKO mice (**Figure 7A**). Upon OTUD1 overexpression in NRCMs under Cycloheximide (CHX) treatment, a decrease in the degradation rate of Pgam5 was observed (**Figure 7B**). We then assessed the role of Otud1 on Pgam5 ubiquitination and observed that Otud1 was able to deubiquitinate Pgam5 at the K63 ubiquitin chain (**Figure 7C-D**). Otud1 expression level manipulation through overexpression or knockout resulted in a decrease or increase in the ubiquitin status of Pgam5, respectively (**Figure 7E-F**). Additionally, the introduction of an active site mutation (C320A) in OTUD1 had no discernible impact on PGAM5 ubiquitination (**Figure 7G**). The impact of Otud1 on Pgam5 protein stability was no longer observed after the mutation (C294S) of mouse Otud1's active site (**Figure 7H**).

Therefore, our findings showed that OTUD1 interacts with PGAM5 and promotes its ubiquitination and degradation.

### OTUD1 promotes Cardiac Hypertrophy in an ASK1-Dependent Manner

To investigate the potential role of Otud1 in promoting pathological cardiac hypertrophy through the phosphorylation of Ask1, adenoviruses (Ad-shOtud1 and Ad-Ask1) were employed to infect NRCMs. The results demonstrated that Ask1 overexpression led to the enlargement of NRCMs (**Figure S8A**), upregulation of hypertrophy-related genes (**Figure S8B**), elevated inflammatory factors expression in the cell supernatant (**Figure S8C**), and MAPK pathway activation (**Figure S8D**).

**Figure 8:**
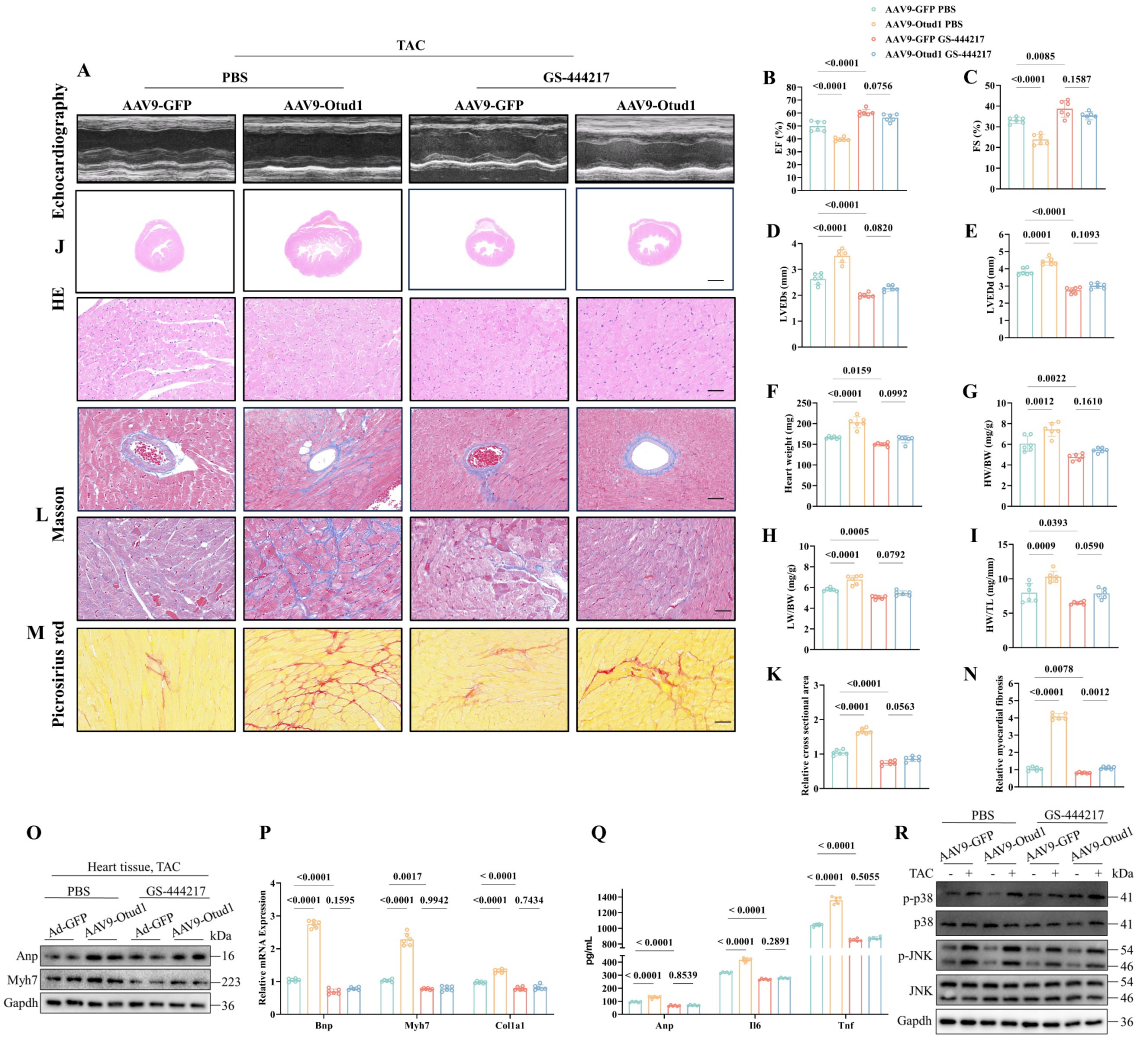
The promotion effect of Otud1 overexpression *in vivo* could be reversed by Ask1 inhibition. (A) Representative M-mode echocardiographic images from each group in mice (n=6). (B) Assessments of ejection fraction (EF) in AAV9-GFP and AAV9-Otud1 TAC mice treated with PBS or GS-444217 at 4 weeks(n=6). (C) Assessments of fraction shortening (FS) in AAV9-GFP and AAV9-Otud1 TAC mice treated with PBS or GS-444217 at 4 weeks (n=6). (D) Assessments of left ventricular end-systolic dimension (LVEDs) in AAV9-GFP and AAV9-Otud1 TAC mice treated with PBS or GS-444217 at 4 weeks (n=6). (E) Assessments of left ventricular (LV) end-diastolic dimension (LVEDd) in AAV9-GFP and AAV9-Otud1 TAC mice treated with PBS or GS-444217 at 4 weeks(n=6). (F) Assessments of heart weight (HW) in AAV9-GFP and AAV9-Otud1 TAC mice treated with PBS or GS-444217 at 4 weeks (n=6). (G) Assessments of HW/body weight (BW) ratios in AAV9-GFP and AAV9-Otud1 TAC mice treated with PBS or GS-444217 at 4 weeks (n=6). (H) Assessments of lung weight (LW)/BW ratios in AAV9-GFP and AAV9-Otud1 TAC mice treated with PBS or GS-444217 at 4 weeks (n=6). (I) Assessments of HW/tibia length (TL) ratios in AAV9-GFP and AAV9-Otud1 TAC mice treated with PBS or GS-444217 at 4 weeks (n=6). (J) Representative images of hematoxylin-eosin (HE) staining of LV cross sections in the hearts of AAV9-GFP and AAV9-Otud1 TAC mice treated with PBS or GS-444217 at 4 weeks (n=6). Scale bar, 1000 μm for the top set and 50 μm for the bottom parts. (K) Relative cross-sectional areas from the hearts of AAV9-GFP and AAV9-Otud1 TAC mice treated with PBS or GS-444217 at 4 weeks. (L) Representative images of Masson staining of cross-sections in the hearts of AAV9-GFP and AAV9-Otud1 TAC mice treated with PBS or GS-444217 at 4 weeks (n=6). Scale bar, 50 μm. (M) Representative images of picrosirius red staining of cross-sections in the hearts of AAV9-GFP and AAV9-Otud1 TAC mice treated with PBS or GS-444217 at 4 weeks (n=6). Scale bar, 50 μm. (N) Relative cardiac fibrosis from the hearts of AAV9-GFP and AAV9-Otud1 TAC mice treated with PBS or GS-444217 at 4 weeks(n=6). (O) Representative western blotting results of Myh7 and Anp in hearts from AAV9-GFP and AAV9-Otud1 TAC mice treated with PBS or GS-444217 at 4 weeks. The experiment was repeated three times. (P) Real-time qPCR analysis of Bnp, Myh7, Col1a1 in heart tissues from AAV9-GFP and AAV9-Otud1 TAC mice treated with PBS or GS-444217 at 4 weeks(n=6). (Q) The content of Anp, Il-6, Tnf-a in plasma from AAV9-GFP and AAV9-Otud1 TAC mice treated with PBS or GS-444217 at 4 weeks (n=6). (R) Representative western blotting results of p-p38, p38, p-JNK, JNK in hearts from AAV9-GFP and AAV9-Otud1 TAC mice treated with PBS or GS-444217 at 4 weeks. The experiment was repeated three times.

Then, we proceeded to investigate the *in vivo* function of the Otud1-Ask1 axis. To accomplish this, we employed AAV9-Otud1 and ASK1 inhibitor (GS-444217) treatments in TAC mice. Notably, our findings from echocardiography demonstrated that Ask1 inhibition effectively mitigated the exacerbating effects of Otud1 on cardiac hypertrophy (**Figure 8A-E**). Furthermore, the HW, HW/BW, LW/BW, and HW/TL of GS-444217-treated mice were observed to be significantly lower compared to those of AAV9-Otud1 mice (**Figure 8F-I**). Ask1 inhibition was found to have the potential to reverse various parameters that were elevated due to Otud1 overexpression. These parameters include heart size (**Figure 8J-K**), cardiac fibrosis (**Figure 8L-N**), hypertrophy gene markers (**Figure 8O-P**), inflammatory factors (**Figure 8Q**), and the MAPK signal pathway (**Figure 8R**). These results indicate that ASK1 mediates the exacerbation role of OTUD1 in cardiac hypertrophy.

## Discussion

Despite significant advancements in the identification of potential molecular targets and signaling pathways within the realm of cardiac hypertrophy 34-37, the existing clinical pharmacological strategies for managing this condition remain unsatisfactory.

To the best of our knowledge, this is the first study to demonstrate that OTUD1 promotes pathological cardiac hypertrophy by upregulating ASK1 phosphorylation. In this study, we found OTUD1 upregulation in heart failure samples from patients at the single-cell level. The expression of Otud1 was also significantly upregulated in TAC-induced myocardium and Ang II-stimulated NRCMs. Our research demonstrated that OTUD1 upregulation was a direct consequence of the METTL3-mediated translation of m6A-methylated OTUD1 mRNA in a YTHDF1-dependent manner. We demonstrated that Otud1 promoted cardiac hypertrophy by stabilizing Pgam5 protein expression, leading to elevated Ask1 phosphorylation. These results demonstrate that Otud1 exacerbates cardiac hypertrophy and could potentially offer therapeutic approaches for pathological cardiac hypertrophy.

DUBs can reverse the ubiquitination process, prevent substrate protein degradation, and participate in cell signaling regulation, protein-protein interactions, and so on 38. The elucidation of the mechanisms by which DUBs perform their functions in cardiac hypertrophy holds the potential to identify novel therapeutic targets. UCHL1 was upregulated in hypertrophic and failing hearts and promoted cardiac hypertrophy by stabilizing EGFR 37. USP2 was downregulated in heart tissues subjected to press overload 39. Ye, B et al. 40 confirmed that USP25 inhibited cardiac hypertrophy by deubiquitinating and stabilizing SERCA2a. OTUD1 has been reported to be involved in inflammatory responses 16, 19, 41, innate immunity 17, drug resistance in cancer 18. OTUD1 was also found to be elevated in vascular endothelium under Ang II stimulation. OTUD1 downregulation attenuated vascular remodeling, collagen deposition, and EndMT by targeting SMAD3 20. In this study, via RNA sequencing, we revealed that Otud1 upregulated genes were associated with cardiac hypertrophy, Apoptosis, inflammatory responses, and protein process.

Increasing amounts of evidence indicate that the involvement of m6A-mediated mRNA modification is vital in cardiovascular diseases 23, 24, 42, 43. A previous study reported that METTL3-mediated m6A modification promotes cardiac hypertrophy by regulating mRNA of MAP3K6, MAP4K5, and MAPK14 25. In this study, we demonstrated that METTL3 facilitated the translation of m6A-methylated OTUD1 mRNA through a YTHDF1-dependent pathway. However, there exists additional m6A writers (METTL14, WTAP4, KIAA14295, RBM15/15B6, and ZC3H137) and erasers (FTO and ALKBH5). Therefore, additional research is needed to determine if other readers and erasers have separate functions in the control of OTUD1 gene expression.

The significance of cell apoptosis in cardiac hypertrophy is widely recognized 44-46. ASK1, also known as MAP3K5, is a member of the MAP kinase family that is involved in the mitogen-activated protein kinase pathway 47. ASK1 exaggerates pathological cardiac hypertrophy 48, 49 and activates JNK and p38 MAPK signaling in cardiovascular diseases 50. Our data showed the significant activation of Ask1 in Otud1-overexpressed hearts, alongside increased phosphorylation of p38 and JNK. This suggests the activation of Ask1-p38/JNK signaling pathways contributes to Otud1-induced cardiac hypertrophy.

The majority of OTU family members possess the activity of deubiquitinating enzymes and takes part in protein posttranslational modification 51. Several OTU family members have been reported to stabilize TopBP1 52, GRP78 53, MYC 54, SLC7A11 14 to regulate cell proliferation, development and death by a ubiquitin proteasome-dependent pathway.

One previous study indicated that Pgam5 functions as an important regulator of Ask1 phosphorylation, acting as a protein serine/threonine phosphatase 55. The binding of Pgam5 and Otud1 was validated by both exogenous and endogenous immunoprecipitation. LFHP-1c, one novel PGAM5 inhibitor, was reported to prevent blood-brain barrier disruption from ischemia 56. The efficacy and safety of PGAM5 inhibitors in the treatment of cardiac hypertrophy was validated in this study, highlighting the need for further investigation.

Notably, two recent publications have explored the role of OTUD1 in cardiovascular contexts. The first study demonstrated that OTUD1 promotes pathological cardiac remodeling and heart failure by targeting STAT3 in cardiomyocytes. The authors found that OTUD1 deubiquitinates and stabilizes STAT3, thereby enhancing its activity and contributing to adverse cardiac remodeling and dysfunction 57. The second study (doi: 10.1016/j.bbadis.2024.167018) investigated the involvement of OTUD1 in heart failure induced by isoprenaline and myocardial infarction, identifying PDE5A as a critical target. This study showed that OTUD1 deubiquitinates PDE5A, leading to its stabilization and increased cGMP degradation, which exacerbates cardiac hypertrophy and heart failure 58.

Our research advances the understanding of OTUD1's role in cardiac hypertrophy and remodeling by elucidating the specific molecular mechanism involving the METTL3-mediated m6A modification of OTUD1 mRNA. Unlike previous studies, we highlight the post-transcriptional regulation of OTUD1 and its subsequent effects on protein stability and signaling pathways. Specifically, we demonstrate that METTL3-mediated m6A modification enhances OTUD1 mRNA stability, leading to increased OTUD1 protein expression. This upregulated OTUD1 then interacts with and deubiquitinates PGAM5, promoting ASK1 phosphorylation and the activation of the p38/JNK MAPK signaling pathway. Hence, focusing on the Otud1-Pgam5-Ask1 axis could potentially serve as a promising approach to address pathological cardiac hypertrophy.

This study still has several limitations. First, OTUD1 upregulation in human heart failure samples was detected from public single-cell sequencing data rather than clinically acquired data. Second, sex-based differences in cardiac hypertrophy 59 were not taken into consideration. To address this issue, subgroup analyses should be performed in the future. Third, this study did not identify the role of OTUD1 in fibroblasts and immune cells. Lastly, the Ang II-infused mouse model and other etiologies of heart failure (myocardial infarction, infection, chronic kidney disease, and so on) were not evaluated in this study. Further attention should be paid to the role of OTUD1 in these circumstances.

In summary, we reported for the first time that OTUD1 expression was upregulated due to m6A modification and that the OTUD1-PGAM5-ASK1 ternary complex is a critical cardiac hypertrophy promoter. Mechanically, Otud1 directly interacts with Pgam5 and Ask1 to inhibit Pgam5 ubiquitination degradation and subsequently elevate Ask1 phosphorylation. The deubiquitinate enzyme activity of Otud1 is necessary for the role of the Otud1-Pgam5-Ask1 axis in the regulation of cardiac hypertrophy. This study suggests that Otud1 inhibition may be a promising approach to alleviating cardiac hypertrophy. The small molecular antagonist of Otud1 may offer a new therapeutic strategy for the treatment of heart failure.

## Supplementary Material

Supplementary materials and methods, figures and tables.
