# Supplementary material for: METTL3-mediated m6A modification of OTUD1 aggravates press overload induced myocardial hypertrophy by deubiquitinating PGAM5
Source: Int J Biol Sci. 2024 Sep 9;20(12):4908–21. doi: 10.7150/ijbs.95707 (PMC11414395; doi:10.7150/ijbs.95707)
Supplement: Supplementary file 1 — Supplementary materials and methods, figures and tables. [file ijbsv20p4908s1.pdf]

Methods and Supplemental Materials

Human samples collection

Hypertrophic myocardium samples were collected from heart failure patients undergoing pacemaker electrode replacement. Control samples were sourced from arrhythmia patients without heart failure who also underwent pacemaker electrode replacement. All experiments involving human samples were approved by the Ethics Committee of Changhai Hospital of Naval Medical University (Shanghai, China; approval no.CHEC2024-051) and adhered to the principles outlined in the Declaration of Helsinki.

Animals

Male C57BL/6 were purchased from JieSiJie Laboratory Animal Co. Ltd (Shanghai, China). Mice were feed under specific pathogen-free condition in the Laboratory Animal Center, Second Military Medical University. The animal procedures and experiments were approved by Animal Care and Use Committee of Changhai Hospital of Second Military Medical University and were carried out in accordance with the National Institutes of Health Guide for the Care and Use of Laboratory Animals. In this study, pentobarbital sodium via intraperitoneal injection was used for both euthanasia (200 mg/kg) and anesthesia (50 mg/kg).

In vivo randomization and blinding procedures

Using a random number table, we randomize all animal experiments in the present study. All animal experiments were blinded and groups were assigned in a randomized manner. A temporary random number within the weight range was assigned to each mouse. Once mice had been randomly divided into four groups, they were given their permanent numerical designations in the cages, and a cage was randomly selected from a pool of cages for each group.

The data collection and analysis were conducted by two observers who were blinded to the group assignment and treatment of the animals. Male mice were exclusively chosen for all animal experiments in this study. This decision was based on previous research indicating that estrogen and low testosterone levels might confer protection against cardiac fibrosis. By utilizing male mice, we aimed to induce significant tissue failure and cardiac fibrosis through the challenge of transverse aortic constriction (TAC), thereby ensuring robust results.

Cardiac hypertrophy model

Pathological cardiac hypertrophy is conducted by TAC surgery. Mice (8-10 weeks) were anesthetized. The neck skin was cut to expose the trachea and the second rib was cut along the sternum. After the separation of thymus, the transverse aortic arch was exposed, and was ligated with 6-0 silk against a 27G needle. The chest was closed after the removal of needle. Mice in the sham group underwent similar operation without ligation of transverse aortic arch. Four weeks after TAC, heart function was measured by echocardiography as previously described. After euthanasia, the heart, lung and tibia of mice were dissected and measured, and the ratios of heart weight (HW)/body weight (BW) (mg/g), HW/tibia length (TL) (mg/mm) and lung weight (LW)/body weight (mg/g) were calculated. All operations and subsequent analyses were performed in a blinded fashion.

AAV9-Otud1 overexpression mice

The OTUD1 and GFP genes were amplified from cDNA and ligated into the vector pAAV-cTnT-WPRE (OBiO Technology, Shanghai, China), and constructed plasmid was packaged into AAV9 vectors carrying the cardiac troponin T (cTnT) promoter to drive the expression of GFP (pAAV-cTnT-GFP-WPRE) and Otud1 (pAAV-cTnT-GFP-P2A-Otud1-3xFLAG-WPRE). Viral solution (7.5

× 10^11 vg/ml, 200 µl/mouse) was injected via the tail vein after the mice were anaesthetized. Two weeks after the AAV9 injection, the animals were randomly assigned to either a sham operation group or a TAC group for another four weeks. Surgeries and subsequent analyses were performed in a blinded fashion.

Echocardiography

Mice were anesthetized with 2% isoflurane and transthoracic echocardiography (30 MHz phased-array probe connected to VEVO LAZR-X system) was used to detect cardiac function. Left ventricular (LV) M-mode imaging was performed in parasternal short-axis view at the level of the papillary muscles. LV dimensions were measured at end-diastole (LVEDd) and end-systole (LVEDs). LV fractional shortening (FS) was calculated as (LVEDd - LVEDs)/LVEDd × 100%. LV ejection fraction (EF) was calculated as $(LVEDd^3 - LVEDs^3)/LVEDd^3 \times 100\%$.

Administration of ASK1 inhibitor GS-444217

TAC mice were randomized to be administered vehicle only or GS-444217 (0.2% in chow) for 4 weeks.

Administration of METTL3 inhibitor STM2457

TAC mice were randomized to be administered vehicle only or STM2457 (50 mg/kg) daily for 4 weeks.

Administration of PGAM5 inhibitor LFHP-1c

TAC mice were randomized to be administered vehicle only or STM2457 (3 mg/kg) daily for 4 weeks.

Hematoxylin-Eosin staining

Paraffin-embedded sections of heart were dewaxed, dehydrated, rinsed in distilled water. Then, the

sections were stained with hematoxylin solution for 3 min, washed with running tap water for 5 min and counterstained in eosin alcoholic solution for 1 min. At last, the sections were dehydrated in increasing concentrations of ethyl alcohol and cleared in xylene for 2 min. A microscope was used to taken pictures which were evaluated by two investigators blinded to the grouping.

Masson's trichrome staining

Masson's Trichrome Stain Kit (G1340, Solarbio, Beijing, China) was used to perform Masson's trichrome staining. Briefly, after deparaffinization and rehydration, the sections were stained in a Weigert iron hematoxylin solution for 5 min, differentiated with acidic ethanol solution for 10 s and treated with Masson bluing solution for 3 min. Then, the sections were incubated with Ponceau S solution for 5 min and rinsed with 1% acetic acid for 1 min. Next, they were placed in 2.5% phosphotungstic acid solution for 10 min and rinsed with 1% acetic acid. Finally, the tissue sections were immersed in aniline blue solution, dehydrated in ethanol, cleared in xylene and sealed with resinene.

Picrosirius red staining

Heart sections were dewaxed in xylene for 15 min (twice), dehydrated in gradient ethanol and washed with running tap water for 30s. Then, the sections were immersed in 0.1 % picrosirius red staining for 90 min and washed in running tap water for 10s. After dyeing, sections were treated with 0.5% acetic acid for 20 s, dehydrated in ethanol and cleared in xylene. The sections were sealed with resinene before light microscope observation.

Histological analysis

Image-Pro Plus (Version 6.0) was used to obtain images from sections stained with hematoxylin-eosin, Masson and Picrosirius red, and the cross-sectional areas of cardiomyocytes and fibrotic areas

were evaluated.

Primary cardiomyocytes isolation and cell treatment

Primary neonatal rat cardiac myocytes (NRCMs) were isolated from hearts of Sprague-Dawley rats within 3 days. Briefly, hearts were minced, digested in 0.2 % Collagenase II (LS0004176, Worthington Bio, USA) for 15 min. The supernatant was obtained and the sediment was redigested for several times until disappeared. After removing the fibroblasts, NRCMs were cultured in High Glucose DMEM (11965092, Gibco) containing 10 % fetal bovine serum (A5669801, Gibco) and 1 % penicillin/streptomycin (C0222, Beyotime Biotechnology, Shanghai, China). NRCMs were infected with specified adenoviruses at a multiplicity of infection of 50 for 24 hours, followed by stimulated with 10μM Ang II (HY-13948, MedChemExpress, USA) for 48 hours.

Human Embryonic Kidney 293 cells (HEK293) and human cardiomyocyte cell line (AC16) were cultured in High Glucose DMEM with 10% fetal bovine serum and 1% Penicillin/streptomycin. Corresponding plasmids were transfected to HEK293 or AC16 with jetPRIME® transfection reagent (101000027, Polyplus Transfection®, France) according to manufacturer's instruction.

Immunofluorescence staining

NRCMs were fixed with 4 % paraformaldehyde at room temperature for 20 min, washed with PBS for 3 min (three times), incubated with 0.2% Triton-X 100 (93443, Merck KGaA, Darmstadt, Germany) for 15 min at room temperature, blocked in blocking buffer (P0260, Beyotime Biotechnology, Shanghai, China) for 15 min. Then, NRCMs were incubates with primary antibody at 4℃ overnight and corresponding secondary antibody for 1h at room temperature. DAPI (C1005, Beyotime Biotechnology, Shanghai, China) was used to stain cell nucleus . Images were obtained via fluorescence microscope.

Western blot analysis

The RIPA buffer (P0013C, Beyotime Biotechnology, Shanghai, China) was used to extract protein from heart tissues and myocardial cells. BCA Protein Assay Kit (P0012, Beyotime Biotechnology, Shanghai, China) was used to detect protein concentration. After that, equal amount of protein was added and separated by 10% SDS-PAGE (PG112, Epizyme Biotech, Shanghai, China). Next, protein was transferred to PVDF membrane (88520, Thermo Fisher Scientific Inc, Carlsbad, CA, USA). Then, the membrane was blocked with 5% nonfat milk for one hour and incubated with primary antibody overnight at 4℃. After that, the membrane was rinsed with TBST solution for 15 min (three times) and incubated with HRP-conjugated secondary antibody at room temperature for one hour. Finally, the membrane was immersed with the chemiluminescence (ECL) reagent and the relative expression of protein was detected by an ECL system. The primary antibodies used was showed in **Supplementary Table S1.**

Quantitative real-time PCR (qPCR)

TRIzol reagent (15596026, Thermo Fisher Scientific Inc, Carlsbad, CA, USA) was used to isolated total RNA from NRCMs and heart tissue according to manufacturer's instructions. The first-strand cDNA was synthesized from 2 μg of total RNA by PrimeScript RT Master Mix (RR036A, TaKaRa, Japan). The mRNA expression levels of related genes were analyzed on a LightCycler® 480 Ⅱ system (Roche, Basel, Switzerland) using TB Green (RR820Q, TaKaRa, Japan). The levels of detected mRNA were calculated by $2^{-\Delta\Delta Ct}$ method and normalized to the amount of endogenous glyceraldehyde-3-phosphate dehydrogenase (GAPDH). The sequences of the qPCR primers were listed in **Supplementary Table S2.**

N6-methyladenosine (m6A) dot blot

The isolated total RNA was heated at 95°C for 3 min to destruct the secondary structure. Then, the RNA samples were transferred to Amersham Hybond-N$^+$ membranes (YA1760, Solarbio Life Science, Beijing, China) followed by ultraviolet cross-linking for 60 min. Then, the membranes were blocked in 5% nonfat milk (dissolved in TBST) for 60 min and incubated with m6A antibody (A19841, ABclonal Technology, Wuhan, China) at 4°C overnight. Next, the membranes were incubated with HRP-conjugated secondary antibody and visualized by chemiluminescence method. To ensure consistency among different groups, the membrane was stained with 0.1% methylene blue solution (7220-79-3, Solarbio Life Science, Beijing, China).

RNA immunoprecipitation coupled with quantitative reverse transcription polymerase chain reaction (RIP-qPCR)

RIP-qRT-PCR was performed using Magna RIP™ Kit (17-704, Merck KGaA, Darmstadt, Germany) according to manufacturer's instruction. Briefly, after specified treatment, cells (2 × 10^7 cells per RIP reaction) were harvested and lysed in a buffer containing RNase and protease inhibitor. Then, cell lysis was treated with DNase and added with indicated antibody and corresponding IgG for overnight at 4°C overnight. The protein/RNA complexes were hooked by magnetic protein A/G beads. Finally, the coprecipitated RNA was isolated and reverse transcribed using the protocol described above followed by qRT-PCR. The primers used for m6A RIP-qPCR analysis was listed in **Supplementary Table S3.**

m6A RNA Methylation Quantification

The overall methylation m6A content was detected using the m6A RNA Methylation Assay Kit (Abcam, MA, USA), following the manufacturer's instructions. In brief, 200 ng of total RNA was added to each reaction, along with the diluted capture antibody, detection antibody solution, and

enhancer solution. The m6A level was quantified using colorimetry, with the absorbance of each reaction measured at 450 nm.

RNA stability

NRCMs were infected with targeted or control adenovirus and subjected to actinomycin D treatment (5 μg/mL, MCE). At indicated time points, cells were collected and total RNA was extracted using the miRNeasy Kit (217084, Qiagen). The half-life ($t_{1/2}$) of mRNA was analyzed and calculate by qRT-PCR as described above.

Plasmid, siRNA and recombinant adenoviruses construction

Plasmids encoding full-length and truncated OTUD1, ASK1, PGAM5 were cloned from cDNAs and ligated to pcDNA3.1 vector (OBiO Technology, Shanghai, China). Overexpression plasmids for METTL3, YTHDF1, Ubi-WT, Ubi-K48, UBi-K63 and siRNA for METTL3, YTHDF1, YTHDF2 and YTHDF3 were constructed by GenePharma (Shanghai, China).

The recombinant adenoviral vector pcADV-EF1-mNeonGreen-CMV-MCS-3xFLAG (OBiO Technology, Shanghai, China) and pADV-U6-shRNA-CMV-EGFP (OBiO Technology, Shanghai, China) were used to construct Ad-Otud1, Ad-shOtud1, Ad-Pgam5, Ad-shPgam5, Ad-Ask1, Ad-shAsk1 and corresponding negative control.

The recombinant adeno-associated virus, including pAAV-cTNT-GdGreen-P2A-Otud1-WPRE (AAV9-Otud1), pAAV-cTNT-GdGreen-P2A-Ask1-WPRE (AAV9-Ask1) were constructed by OBiO Technology (Shanghai, China). The primer sequences used for plasmids construction are listed in **Supplementary Table S4**.

Coimmunoprecipitation (Co-IP) and liquid chromatography-tandem mass spectrometry (LC-MS/MS)

To investigate the underling mechanisms of OTUD1 induced pro-hypertrophic effects, Co-IP was carried out using commercially available kit (abs955, Absin Biotechnology, Shanghai, China). NRCMs or 293 cells were washed with PBS. Heart tissues were treated with liquid nitrogen and ground into powder. The lysis buffer with 1 mM PMSF (abs9146, Absin Biotechnology, Shanghai, China) was added to cells or tissue powder at 4℃ for 20min. The lysate were subjected to ultrasonic crushing (FB50220, Thermo Fisher) for 5s (3 times, 10s interval, 40% max power) on the ice and centrifuged (14000 g, 4℃, 10 min) to obtain supernatant. The protein concentration was detected by using BCA assay kit (P0012, Beyotime Biotechnology, Shanghai, China). For removing the non-specific binding proteins, a total of 500μl (1 ug/μl) protein was added with 5 μl Protein A and Protein G agarose beads, and incubated at 4℃ for 60 min on a rotating machine (20 rpm). The newly acquired supernatant were transferred to new centrifugal tubes. For IP group, 1 μg targeted monoclonal antibody was used. For Input group, 1 μg IgG was used. After antigen-antibody combination (4℃, 12h, 20rpm), 5 μl Protein A and Protein G agarose beads were added to link antibody-proteins complex for 2 h at 4 °C with rotation at 20 rpm. After that, the supernatant and beads-antibody-proteins complex were separated by centrifugation (4℃, 1 min, 12000g) followed by three times washing steps (4℃, 1 min, 12000g). The sediments were resuspended with 20 μl SDS buffer, boiled at 100℃ for 5 min and centrifugated for 1 min at 4℃at 12000g. Finally, the supernatants were SDS-PAGE analysis and immunoblotting described above.

For LC-MS/MS, the immunoprecipitation complex was separated by SDS-PAGE and then set to Wuhan Metware Biotechnology (Wuhan, China) for further analysis.

Ubiquitination assays

After pretreatment, cultured NRCMs, 293 cells or heart tissues was subjected to lysis and IP assays

with indicated antibodies, followed by western blot.

Single cell RNA sequencing analysis

The scRNA datasets of the human heart (GSE161470, GSE145154, and GSE161153) were obtained from the publicly available GEO database (https://www.ncbi.nlm.nih.gov/geo/). Subsequently, four left ventricular control samples from GSE161470, three left ventricular HF samples from GSE145154, and one left ventricular sample with HF from GSE161153 were selected for further analysis. This analysis was conducted using R (version 4.3.1) and the Seurat package (version 4.1.1). To form large gene expression matrices, the "merge" function was employed. Cells meeting the criteria of having more than 500 genes, less than 5,000 genes, and less than 20% mitochondrial genes were retained for subsequent analysis. Gene expression lists were normalized using the “NormalizeData” function and further scaled. Subsequently, the “vst” method was used for each sample to identify 2,000 highly variable genes. According to the highly variable genes, principal component analysis was applied to identify significant principal components (PCs), which were visualized using the ElbowPlot function. Since scRNA data were collected from three different research groups, the “Harmony” package (version 0.1.0) was used to correct the batch effect. We employed a sample of 20 PCs to conduct UMAP analysis. The classification of fifteen cell clusters was accomplished through the utilization of the "FindClusters" function, employing a resolution of 0.5. To identify DEGs within each cell cluster, we employed the "FindAllMarkers" function with a threshold of 0.25. The annotation of cell types was performed by considering the top five DEGs for each cell cluster, as well as referencing a previously published article [23] and utilizing the CellMarker database (http://bio-bigdata.hrbmu.edu.cn/CellMarker/).

RNA transcriptome sequencing

After infection of Ad-Otud1 and Ad-GFP, the total RNA was isolated from NRCMs (three independent samples per group) and sent to Wuhan Metware Biotechnology (Wuhan, China) for RNA sequencing, including RNA detection, library construction, and computer sequencing. After removing of reads with low quality, clean reads were mapped to the UCSC rn6 genomes using HISAT2 software (version 2.2.1) and transferred to the BAM format using samtools (version 1.6). Then, the count matrix was created by featureCounts (version 2.0.3) and input into R (version 4.2.3). The limma package (version 3.54.2) was used to identify differentially expressed genes ($P < 0.05$ and $|\log FC| > 1$). The online data of RNA-seq is available on GEO database (https://www.ncbi.nlm.nih.gov/geo/query/acc.cgi?acc=GSE241809).

Gene set enrichment analysis (GESA)

R package "clusterProfiler" (Version 3.8.0) was used to implement GSEA. The gene set collection "c2.cp.kegg.Hs.symbols.gmt" was downloaded from Human Molecular Signatures Database (https://www.gsea-msigdb.org/gsea/msigdb). Gene sets with P value < 0.05 were considered statistically significant.

Enzyme-linked immunosorbent assay

The NRCMs supernatant concentration of Il-6 (EK0412, BOSTER, Wuhan, China) and Tnf-a (EK0526, BOSTER, Wuhan, China) were detected by ELISA kits according to the manufacturers' instructions.

The mice plasma concentration of Anp (NBP2-66733, Novus Biologicals, USA), Il-6 (EK0411, BOSTER, Wuhan, China) and Tnf-a (EK0527, BOSTER, Wuhan, China) were detected by ELISA kits according to the manufacturers' instructions.

Statistical analyses

Data were analyzed by GraphPad Prism 9 (GraphPad Software, Inc., CA, USA). All experiments were performed in triplicate unless stated otherwise. Values are presented as mean ± SD or median ± interquartile range. The Shapiro-Wilk test was used to evaluate normal distribution of data. For two group comparison, unpaired two-tailed Student's t-test (normal distribution) and Mann-Whitney test (skewed normal distribution) were used. For three or more groups comparisons, one-way ANOVA followed by Tukey post hoc analysis (normal distribution, homogeneity of variance), two-way ANOVA followed by Bonferroni post hoc test (normal distribution, homogeneity of variance, multi-factors), one-way ANOVA followed by Tamhane T2 post hoc analysis (normal distribution, heteroscedasticity) and Kruskal-Wallis test followed by Dunn post hoc analysis (skewed normal distribution) were used. The exponential decay equation models for mRNA half-life were developed using GraphPad Prism 9. The statistical tests used for the data in each figure are listed in the related legends. The sample size of mice in each experiment is indicated in related legends and determined based on previous studies [10,35]. No statistical method was used to predetermine the sample size.

Supplementary Table S1 Primary antibodies used in this study

**Source**	**Antibody**	**Cat No**
Abcam	OTUD1	ab122481
Proteintech	α-actinin	11313-2-AP
Proteintech	ANP	27426-1-AP
ABclonal	MYH7	A4963
Proteintech	GAPDH	60004-1-Ig
ABclonal	m6A	A22411
Proteintech	IgG	B900620
Proteintech	METTL3	15073-1-AP
Proteintech	YTHDF1	17479-1-AP

Proteintech	YTHDF2	24744-1-AP
Proteintech	YTHDF3	25537-1-AP
Abcam	PGAM5	ab244218
Proteintech	PGAM5	28445-1-AP
Proteintech	ASK1	67072-1-Ig
Proteintech	ASK1	28201-1-AP
CST	p-ASK1	3765S
Abcam	ERK1/2	ab184699
Abcam	p-ERK1/2	ab214036
CST	ERK5	3372S
CST	p-ERK5	3371S
Proteintech	p38	14064-1-AP
Proteintech	p-p38	28796-1-AP
Proteintech	JNK	17572-1-AP
Proteintech	p-JNK	80024-1-RR
Proteintech	Flag tag	66008-4-Ig
Proteintech	Flag tag	20543-1-AP
Proteintech	His tag	66005-1-Ig
Proteintech	His tag	10001-0-AP
Proteintech	HA tag	66006-2-Ig
Proteintech	HA tag	51064-2-AP
Proteintech	Myc tag	60003-2-Ig
Proteintech	Ubiquitin	80992-1-RR

Supplementary Table S2 The primer sequences for qPCR

Gene	Sequences (5’-3’)	
Rat Otud1	Forward	TGGCTTAGTTGGCTCAGCAA
	Reverse	CGTCGCGTTTTCTCTGCATT
Rat Anp	Forward	AAAGCAAACTGAGGGCTCTGCTCG
	Reverse	TTCGGTACCGGAAGCTGTTGCA
Rat Bnp	Forward	TGCCCCAGATGATTCTGCTC
	Reverse	TGTAGGGCCTTGGTCCTTTG
Rat Myh7	Forward	AGTTCGGGCGAGTCAAAGATG
	Reverse	CAGGTTGTCTTGTTCCGCCT
Rat Gapdh	Forward	ACTCTACCCACGGCAAGTTC
	Reverse	TGGGTTTCCCGTTGATGACC
Mouse Otud1	Forward	AGAGGCAGGACAAGTACCTGA
	Reverse	CCCGTACACAGTCTTGCTGAC
Mouse Col1a1	Forward	TGCTAACGTGGTTCGTGACCGT
	Reverse	ACATCTTGAGGTCGCGGCATGT
Mouse Bnp	Forward	GTGACGTTGACATCCGTAAAGA
	Reverse	GCCGGACTCATCGTACTCC

Mouse Myh7	Forward	CAACCTGTCCAAGTTCCGCA
	Reverse	TACTCCTCATTCAGGCCCTTG
Mouse Gapdh	Forward	ATGTGTCCGTCGTGGATCTG
	Reverse	AGTTGGGATAGGGCCTCTCTT

Supplementary Table S3 The primers used for m6A RIP-qPCR analysis.

Gene	Sites	Sequences (5'-3')	
OTUD1	Site 1	Forward	GTGGCGCAGCAGGAATTTG
		Reverse	CCAACGGCCCCAGAATAGAG
OTUD1	Site 2	Forward	TGATCGTCTCCAGGTCGGAT
		Reverse	GCCGTCTGGAATGATGTGGA
OTUD1	Site 3	Forward	TTATCATCGCTGCTGCCCAA
		Reverse	CATGGTAGACACCGTGGGAC
OTUD1	Site 4	Forward	TGATGCTGTATTTGATCACTCCT
		Reverse	CGCGTTTCCTTTGCACTTGA
OTUD1	Site 5	Forward	CAAGTGCAAAGGAAACGCGA
		Reverse	CCCAGGGTGTAAGGTTTTGAGA
OTUD1	Site 6	Forward	GGAGCCAACCTTGGCAGTTA
		Reverse	GACCCAGCAAACCATACAAGC

Supplementary Table S4 The primers used for overexpression plasmids, adenoviruses plasmids

Primer sequences for overexpression plasmids:

Gene	Sequences (5'-3')	
Human METTL3	Forward	CCCAAGCTTATGTCGGACACGTGGAGC
	Reverse	ATTTGCGGCCGCCTATAAATTCTTAGGTTTAGAGA
Human YTHDF1	Forward	CGGAATTCATGTCGGCCACCAGCGTGGACA
	Reverse	TCTAGAGCTCATTGTTTGTTTCGACTCTGCC
Human OTUD1 (WT)	Forward	CGGAATTCATGCAGCTCTACAGCAGCGTCTG
	Reverse	TCTAGAGCTCAAGAGCATGCATTTTGTTCA
Human OTUD1 (C320A)	Forward	CGGAATTCATCATTCCAGACGGCAACG

		CCCTCTACCGAGCTGTCAGC
	Reverse	TCTAGAGCGCTGACAGCTCGGTAGAGG GCGTTGCCGTCTGGAATGAT
Human OTUD1 (ΔAla)	Forward	CGGAATTCATGCAGCTCTACAGCAGCG TCT
	Reverse	TCTAGAGCTCAAGAGCATGCATTTTGTT C
Human OTUD1 (ΔLinker)	Forward	CGGAATTCATGCAGCTCTACAGCAGCG TCTGC
	Reverse	TCTAGAGCTCAAGAGCATGCATTTTGTT C
Human OTUD1 (ΔOTU)	Forward	CGGAATTCATGCAGCTCTACAGCAGCG TCT
	Reverse	TCTAGAGCTCAAGAGCATGCATTTTGT CAAG
Human OTUD1 (ΔUIM)	Forward	CGGAATTCATGCAGCTCTACAGCAGGC TCTGC
	Reverse	TCTAGAGCTCAAGAGCATGCATTTTGC CTTTGC
Human ASK1 (WT)	Forward	ATATGGATCCATGAGCACGGAGGCGGA CGAGG
	Reverse	ACCGGTCGTCAAGTCTGTTTGTTTCGA AAGT
Human ASK1 (Δ1-648aa)	Forward	ATATGGATATGGTGAACACCATTACGCA AG
	Reverse	ACCGGTCGTCAAGTCTGTTTGTTTCGA AAG
Human ASK1 (Δ649-955aa)	Forward	ATATGGATATGAGCACGGAGGCGGACG AGGG
	Reverse	ACCGGTCGTCAAGTCTGTTTGTTTCGA AAGT
Human ASK1 (Δ956-1374aa)	Forward	ATATGGATATGAGCACGGAGGCGGACG AGG
	Reverse	ACCGGTCGTGAAAGAGCTGAAAGCTT AGGT
Human PGAM5 (WT)	Forward	ATGGTACCATGGCGTTCCGGCAGGCGC TGC
	Reverse	TCTAGAGCTCAGGATCGAGTGATCTTG TCG
Human HA-Ubi-WT	Forward	CGGAATTCATGCAGATCTTCGTGAAAA CCC
	Reverse	TCTAGAGCGCCACCCCTCAGACGCAGG ACC

Human HA-Ubi-K48	Forward	CCGGAATTCATGCAGATCTTCGTGAGAACCCT
	Reverse	TCTAGAGCAGCCACCCCTCAGACGCAGGACCA
Human HA-Ubi-K63	Forward	CGGAATTCATGCAGATCTTCGTGAGAACCC
	Reverse	TCTAGAGCGCCACCCCTCAGACGCAGGACCA

Primer sequences for adenoviral plasmids:

Gene	Sequences (5'-3')	
Rat Ad-Otud1 (WT)	Forward	CGGAATTCGCCACCATGCAGCTCTACAGCAGCGTGT
	Reverse	GGATCCCGTCAAGAGCATGCATTCTGTTCG
Rat Ad-Otud1 (Mut1)	Forward	CCGGAATTCGCCACCATGCAGCTCTACAGCAGCGTCT
	Reverse	GGATCCGCGTCAAGAGCATGCATTTTGTTCA
Rat Ad-Otud1 (Mut2)	Forward	CCGGAATTCGCCACCATGCAGCTCTACAGCAGCGTC
	Reverse	GGATCCGCGTCAAGAGCATGCATTTTGTTCA
Rat Ad-Otud1 (Mut3)	Forward	CCGGAATTCGCCACCATGCAGCTCTACAGCAGCGT
	Reverse	TCTAGAGCATCAAGAGCATGCATTTTGTTC
Rat Ad-Otud1 (Mut4)	Forward	CCGGAATTCGCCACCATGCAGCTCTACAGCAGCGTCT
	Reverse	GGATCCGCGTCAAGAGCATGCATTTTGTTC
Rat Ad-Otud1 (C294S)	Forward	CCGGAATTGCCACCCATCATCCCTGACGGTAACTCCCTTTACCGAGCTGTCAGC
	Reverse	TCTAGAGCAGCTGACAGCTCGGTAAAGGGAGTTACCGTCAGGGATGAT
Rat Ad-shOtud1	Forward	CCGGGCAGATGCTGAATGTGAATTTCAAGAGAATTCACATTCAGCATCTGCTTTTTTG
	Reverse	AATTCAAAAAAGCAGATGCTGAATGTGAATTCTCTTGAAATTCACATTCAGCATCTGC
Rat Ad-Ask1	Forward	CGGAATTCCGCCACCATGGGCACCGAAGCCGGCGAGG
	Reverse	TCTAGAGCTCAGGCCCCTTTGTTCCGAAAG
Rat Ad-shAsk1	Forward	CCGGGGTGCAAAGTGAAGAGCAAGTTTCAAGAGAACTTGCTCTTCACTTTGCACCTTTTTTG
	Reverse	AATTCAAAAAAGGTGCAAAGTGAAGAGCAAGTTCTCTTGAAACTTGCTCTTCACTTTGCACC
Rat Ad-Pgam5	Forward	CGGAATTCGCCACCATGGCTTTCCGGCAGGCTCTTCA

	Reverse	TCTAGAGCTCAGGACCGAGTAATCTTGTCT
Rat Ad-shPgam5	Forward	CCGGGCCTGGGACTGAAGTTTAATATTCAAG AGATATTAAACTTCAGTCCCAGGCTTTTTG
	Reverse	AATTCAAAAAAGCCTGGGACTGAAGTTTAAT ATCTCTTGAAGCCTGGGACTGAAGTTTAATA

siRNA sequences:

Gene	sequence
Human METTL3	5′-GCACTTGGATCTACGGAAT-3′
Human YTHDF1	5′-GATACAGTTCATGACAATGA-3′
Human YTHDF2	5′-CTGCCATGTCAGATTCCTA-3′
Human YTHDF3	5′-ATGGATTAAATCAGTATCTAA-3′

Figure S1

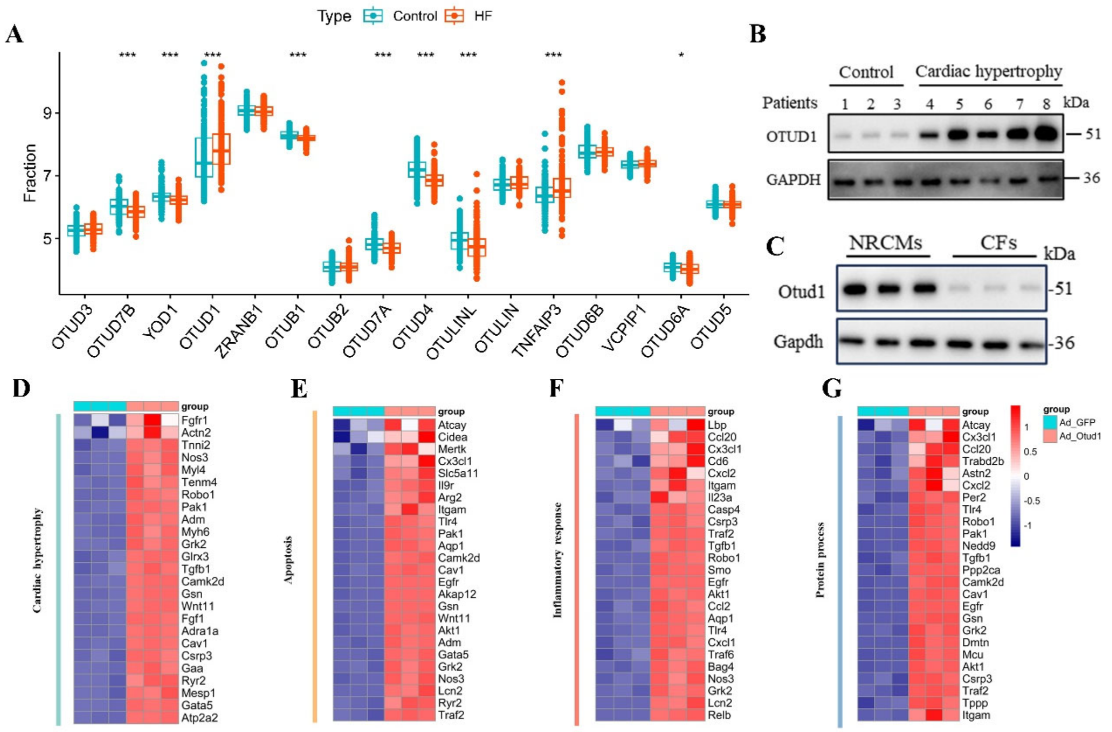

(A) Boxplot showing the expression of genes from OUT family in GSE57338.

(B) The expression of OTUD1 protein in controls and cardiac hypertrophy patients.

(C) OTUD1 was mainly enriched in cardiomyocytes.

(D-G) Heatmaps showing the significantly altered genes related to hypertrophy, Apoptosis, inflammatory response and protein process.

Figure S2

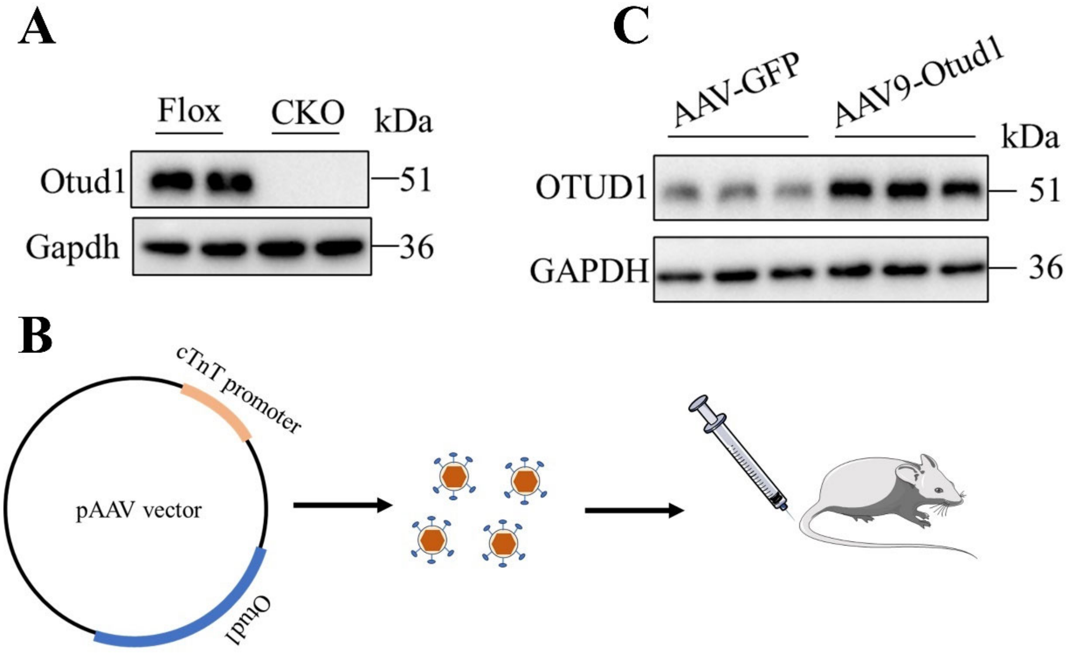

(A) Representative immunoblotting of Otud1 protein in the heart from Flox and Otud1-CKO mice (n=4).

(B) Schematic diagram describing the strategy for the generation of AAV9-Otud1 mice.

(C) Representative immunoblotting of Otud1 protein in the heart from AAV9-GFP and AAV9-Otud1 mice (n=4).

Figure S3

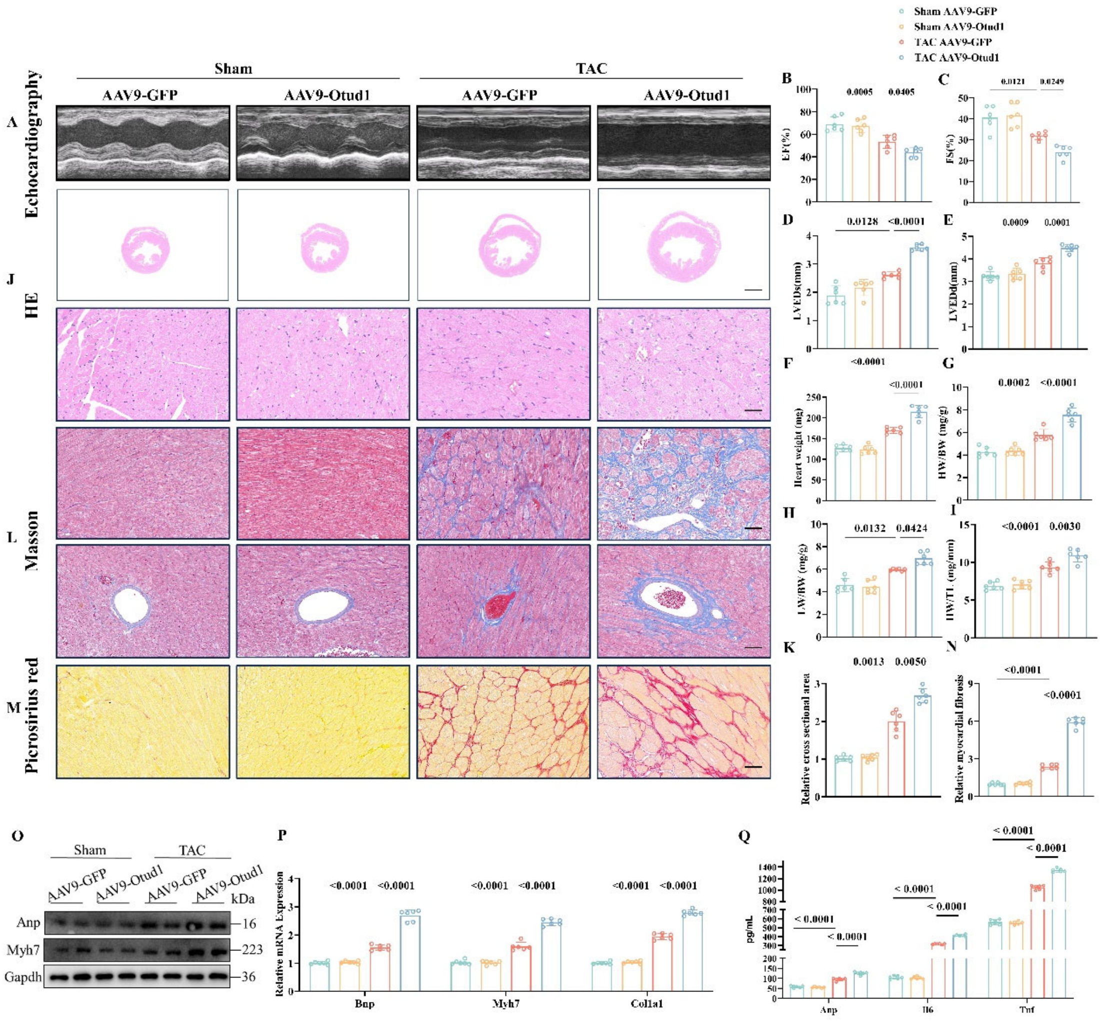

(A) Representative M-mode echocardiographic images from each group in mice (n=6).

(B) Assessments of ejection fraction (EF) in AAV9-GFP and AAV9-Otud1 mice at 4 weeks after Sham or TAC surgery (n=6).

(C) Assessments of fraction shortening (FS) in AAV9-GFP and AAV9-Otud1 mice at 4 weeks after Sham or TAC surgery (n=6).

(D) Assessments of left ventricular end-systolic dimension (LVEDs) in AAV9-GFP and AAV9-Otud1 mice at 4 weeks after Sham or TAC surgery (n=6).

(E) Assessments of left ventricular (LV) end-diastolic dimension (LVEDd) in AAV9-GFP and AAV9-Otud1 mice at 4 weeks after Sham or TAC surgery (n=6).

(F) Assessments of heart weight (HW) in AAV9-GFP and AAV9-Otud1 mice at 4 weeks after Sham or TAC surgery (n=6).

(G) Assessments of HW/body weight (BW) ratios in AAV9-GFP and AAV9-Otud1 mice at 4 weeks after Sham or TAC surgery (n=6).

(H) Assessments of lung weight (LW)/BW ratios in AAV9-GFP and AAV9-Otud1 mice at 4 weeks after Sham or TAC surgery (n=6).

(I) Assessments of HW/tibia length (TL) ratios in AAV9-GFP and AAV9-Otud1 mice at 4 weeks after Sham or TAC surgery (n=6).

(J) Representative images of hematoxylin-eosin (HE) staining of LV cross sections in the hearts of AAV9-GFP and AAV9-Otud1 mice at 4 weeks after Sham or TAC surgery (n=6). Scale bar, 1000 μm for the top set and 50 μm for the bottom parts.
(K) Relative cross-sectional areas from the hearts of in AAV9-GFP and AAV9-Otud1 mice at 4 weeks after Sham or TAC surgery (n=6).
(L) Representative images of Masson staining of cross-sections in the hearts of in AAV9-GFP and AAV9-Otud1 at 4 weeks after Sham or TAC surgery (n=6). Scale bar, 50 μm.
(M) Representative images of picrosirius red staining of cross-sections in the hearts of AAV9-GFP and AAV9-Otud1 mice at 4 weeks after Sham or TAC surgery (n=6). Scale bar, 50 μm.
(N) Relative cardiac fibrosis from the hearts of AAV9-GFP and AAV9-Otud1 mice at 4 weeks after Sham or TAC surgery (n=6).
(O) Representative western blotting results of Myh7 and Anp in hearts from AAV9-GFP and AAV9-Otud1 at 4 weeks after Sham or TAC surgery. The experiment was repeated three times.
(P) Real-time qPCR analysis of Bnp, Myh7, Col1a1 in heart tissues from AAV9-GFP and AAV9-Otud1 mice at 4 weeks after Sham or TAC surgery (n=6).
(Q) The content of Anp, Il-6, Tnf-a in plasma from AAV9-GFP and AAV9-Otud1 mice at 4 weeks after Sham or TAC surgery (n=6).

Figure S4

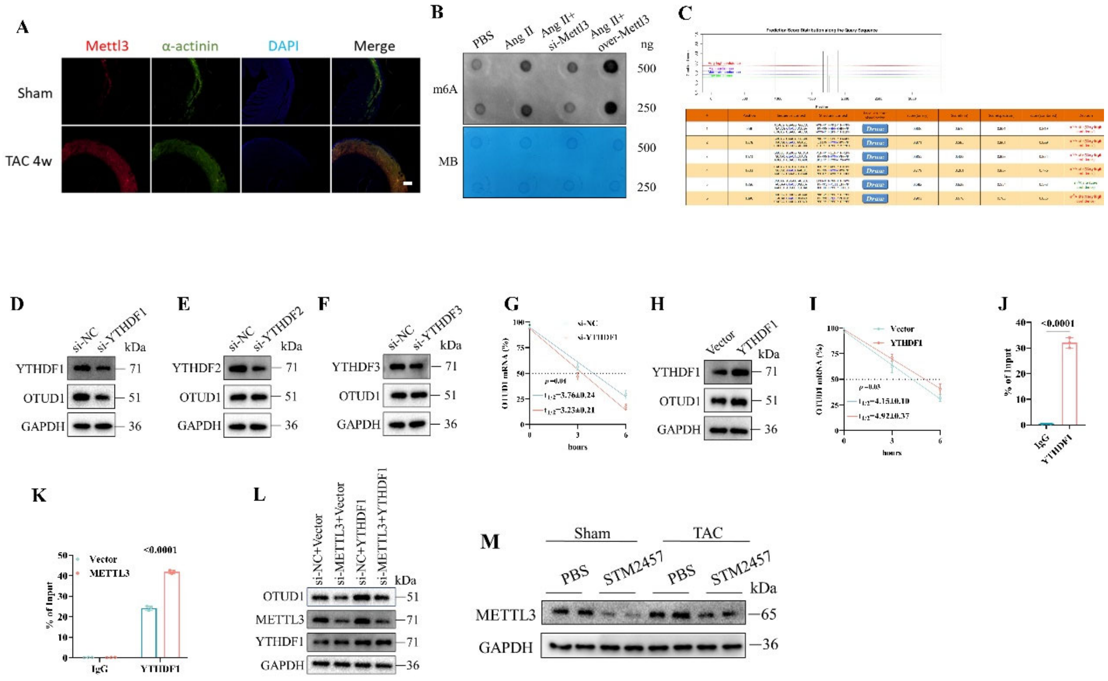

(A) Mettl3 level in heart tissues were determined by immune fluorescence. Scale bar, 100 μm.
(B) m6A dot plot showing the m6A level in four groups. Methylene blue staining served as a loading control.
(C) The predicted m6A motif in OTUD1 mRNA.
(D) OTUD1 protein levels in AC16 transfected with si-YTHDF1. The experiment was repeated

three times.

(E) OTUD1 protein levels in AC16 transfected with si-YTHDF2. The experiment was repeated three times.

(F) OTUD1 protein levels in AC16 transfected with si-YTHDF3. The experiment was repeated three times.

(G) OTUD1 RNA stability in YTHDF1-knockdown AC16 was measured by one-phase decay analysis (n=3).

(H) OTUD1 protein levels in AC16 transfected with YTHDF1 plasmid. The experiment was repeated three times.

(I) OTUD1 RNA stability in YTHDF1 overexpessed AC16 was measured by one-phase decay analysis (n=3).

(J) The interaction between the YTHDF1 protein and OTUD1 mRNA in AC16 was measured by RIP followed by qRT-PCR analysis (n=3).

(K) The interaction between the YTHDF1 protein and OTUD1 mRNA in METTL3-overexpressed AC16 was measured by RIP followed by qRT-PCR analysis (n=3).

(L) Western blot analysis of OTUD1 protein levels in AC16 after transfection with or without si-METTL3, followed by treatment with or without YTHDF1 plasmid. The experiment was repeated three times.

(M) Western blot analysis of cardiac METTL3 protein after treatment with PBS or STM2457 followed by sham or TAC surgery. The experiment was repeated three times.

Figure S5

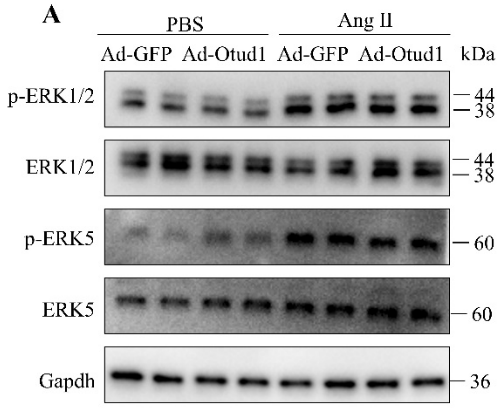

(A) Representative western blotting results of p-ERK1/2, ERK1/2, p-ERK5, ERK5 in NRCMs infected with Ad-GFP, Ad-Otud1 and treated with Ang II (10 μM) or PBS for 48h.

Figure S6

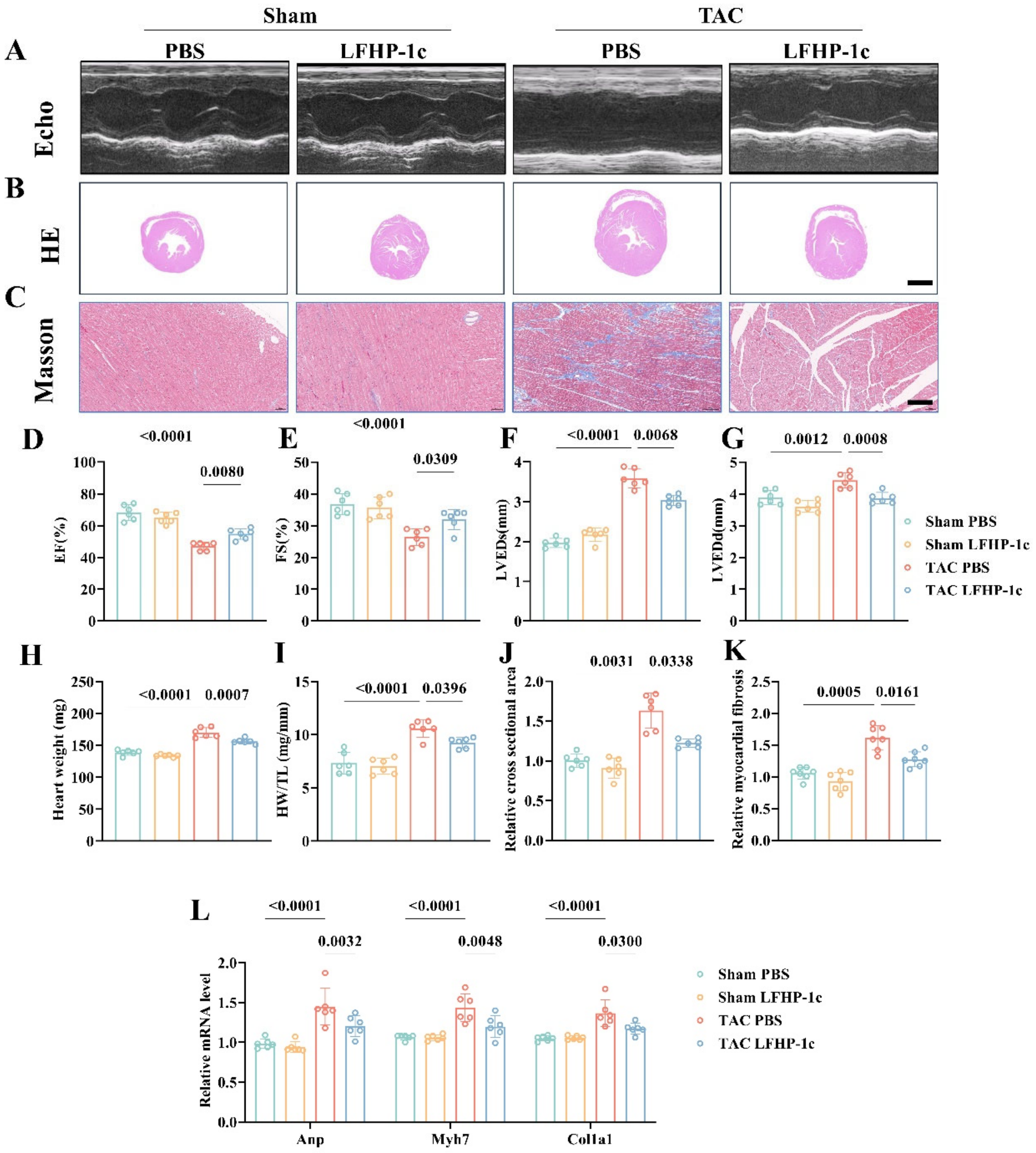

(A) Representative M-mode echocardiographic images from each group in mice (n=6).

(B) Representative images of HE staining of LV cross sections in the hearts from each group (n=6). Scale bar, 1000 μm for the top set and 50 μm for the bottom parts.

(C) Representative images of Masson staining of cross-sections in the hearts from each group (n=6). Scale bar, 50 μm.

(D) Assessments of ejection fraction (EF) in each group (n=6).

(E) Assessments of fraction shortening (FS) in each group (n=6).

(F) Assessments of left ventricular end-systolic dimension (LVEDs) in each group (n=6).

(G) Assessments of left ventricular (LV) end-diastolic dimension (LVEDd) in each group (n=6).

(H) Assessments of heart weight (HW) in each group (n=6).

(I) Assessments of HW/tibia length (TL) ratios in each group (n=6).

(J) Relative cross-sectional areas from the hearts of in each group (n=6).

(K) Relative cardiac fibrosis from the hearts in each group (n=6).

(L) Real-time qPCR analysis of Bnp, Myh7, Col1a1 in heart tissues from PBS and LFHP-1c treated mice at 4 weeks after Sham or TAC surgery (n=6).

Figure S7

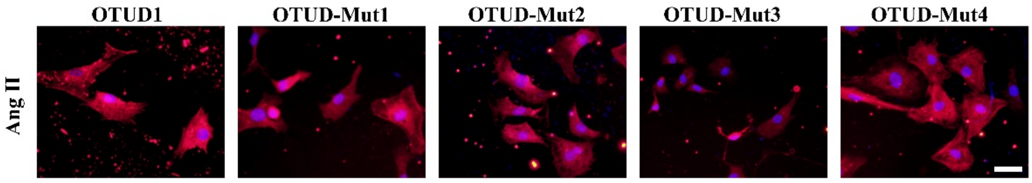

Representative immunofluorescence images of the cell surface area from NRCMs infected with Ad-OTUD1, OTUD1-Mut1, OTUD1-Mut2, OTUD1-Mut3, OTUD1-Mut4 and treated with Ang Ⅱ (10 μM) or PBS for 48 h (n≥50 cells per group). Scale bar, 50 μm

Figure S8

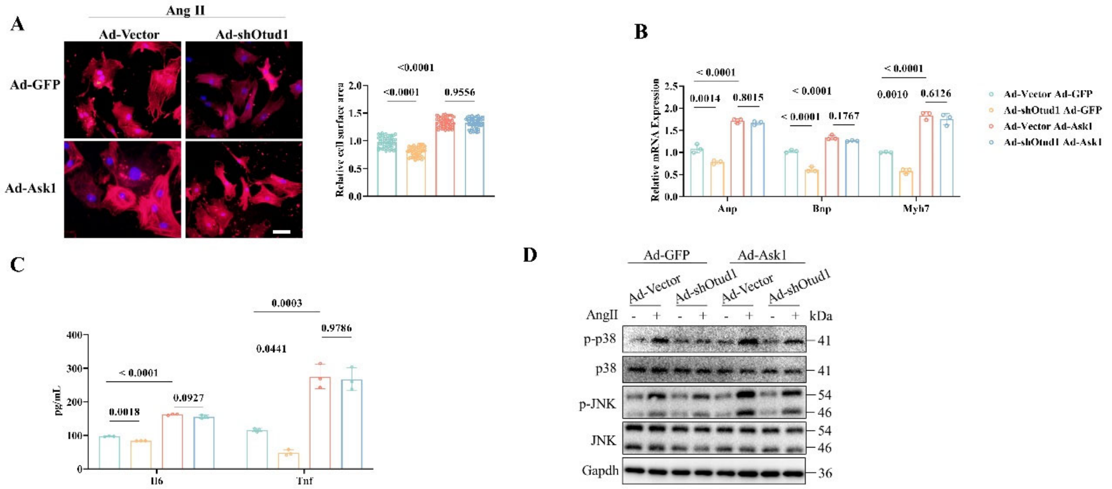

(A) Representative immunofluorescence images of the cell surface area from NRCMs infected with Ad-shOtud1, Ad-Ask1 and treated with AngII (10 μM) or PBS for 48 h (n≥50 cells per group). Scale bar, 50 μm

(B) Real-time qPCR analysis of Anp, Bnp and Myh7 in NRCMs infected with Ad-shOtud1, Ad-Ask1 and treated with AngII (10 μM) or PBS for 48h.

(C)) The content of Il6, Tnf in cell supernatant from NRCMs infected with Ad-shOtud1, Ad-Ask1 and treated with AngII (10 μM) or PBS for 48 h.

(D) Representative western blotting results of p-p38, p38, p-JNK, JNK in NRCMs infected with Ad-shOtud1, Ad-Ask1 and treated with AngII (10 μM) or PBS for 48h.
